# F-Spondin/spon1b Expression Patterns in Developing and Adult Zebrafish

**DOI:** 10.1371/journal.pone.0037593

**Published:** 2012-06-29

**Authors:** Veronica Akle, Emmanuel Guelin, Lili Yu, Helena Brassard-Giordano, Barbara E. Slack, Irina V. Zhdanova

**Affiliations:** 1 Department of Anatomy & Neurobiology, Boston University School of Medicine, Boston, Massachusetts, United States of America; 2 Department of Pathology and Laboratory Medicine, Boston University School of Medicine, Boston, Massachusetts, United States of America; Institut Curie, France

## Abstract

F-spondin, an extracellular matrix protein, is an important player in embryonic morphogenesis and CNS development, but its presence and role later in life remains largely unknown. We generated a transgenic zebrafish in which GFP is expressed under the control of the F-spondin (spon1b) promoter, and used it in combination with complementary techniques to undertake a detailed characterization of the expression patterns of F-spondin in developing and adult brain and periphery. We found that F-spondin is often associated with structures forming long neuronal tracts, including retinal ganglion cells, the olfactory bulb, the habenula, and the nucleus of the medial longitudinal fasciculus (nMLF). F-spondin expression coincides with zones of adult neurogenesis and is abundant in CSF-contacting secretory neurons, especially those in the hypothalamus. Use of this new transgenic model also revealed F-spondin expression patterns in the peripheral CNS, notably in enteric neurons, and in peripheral tissues involved in active patterning or proliferation in adults, including the endoskeleton of zebrafish fins and the continuously regenerating pharyngeal teeth. Moreover, patterning of the regenerating caudal fin following fin amputation in adult zebrafish was associated with F-spondin expression in the blastema, a proliferative region critical for tissue reconstitution. Together, these findings suggest major roles for F-spondin in the CNS and periphery of the developing and adult vertebrate.

## Introduction

The extracellular matrix (ECM) is an essential component of many tissues, providing for structural support and guidance, and affecting signaling and homeostasis [1]. A small family of ECM proteins, called spondins, is evolutionarily well conserved and includes f-spondin, mindin and subcommissural organ (SCO)-spondin [2]. F-spondin was found to be an important player in embryonic morphogenesis in such diverse species as *C. elegans* and rat [3], [4]. It is also expressed in adult tissues and might play diverse roles in the central nervous system (CNS) and the periphery [2], [5]–[8].

The F-spondin molecule consists of about 800 amino acids, and contains an N-terminal domain homologous to the amino terminus of reelin, a spondin domain and six C-terminal thrombospondin repeats [4]. This structure allows F-spondin to affect different processes through binding to the ECM or membrane receptors. For example, during development, the two proteolytic fragments of F-spondin were found to play opposing though complementary roles in guiding the growth of commissural neurons between the floor plate cells and the basement membrane [9]. Thus, whereas the C-terminal fragment binds to the ECM, promoting neuronal outgrowth along the basement membrane beneath the floor plate, the N-terminal fragment binds to several members of the low density lipoprotein receptor family (ApoER2, LRP2/megalin, and LRP4), inhibiting neuronal outgrowth and preventing these neurons from growing through the floor plate [9]. These effects of F-spondin are likely to be conserved, since it is present in the embryonic floor plate in the frog, mouse, chick, and zebrafish [4], [10]–[12]. The expression of F-spondin in other brain regions of embryonic rat or zebrafish and, at lower levels, in the neocortex and hippocampus of adult rats, suggests its broader role in CNS development and maintenance [12], [13]. Indeed, studies *in vitro* found that F-spondin accumulates in the ECM that ensheaths the developing peripheral nerves [14], promotes neurite outgrowth from embryonic hippocampal and commissural neurons [2], [13], and potentiates nerve precursor differentiation [5].

*In vitro* studies also suggest that the effects of F-spondin depend on its concentration and presence in substrate-attached or soluble form [5]. Thus, the exact sites of F-spondin production and its release into the cerebrospinal fluid (CSF) compartments of the brain might determine its availability and efficacy. This is especially interesting in view of recent evidence that F-spondin is a putative ligand for the Alzheimer disease-related amyloid precursor protein (APP), and may exert its neurotrophic effects in part via a specific interaction with APP [15]–[17]. Since F-spondin binds ApoER2 via its thrombospondin domain, and APP via its reelin and spondin domains, a single F-spondin molecule could form a complex with APP and ApoER2 [17]. Indeed, an interaction between F-spondin and APP and/or ApoEr2, affecting the APP-downstream signaling molecule disabled-1 (DAB-1), has been suggested as one of the mechanisms by which F-spondin might control neuron survival and neuroblast migration [16], [18].

Together, these findings underscore a need for systematic characterization of F-spondin expression patterns in the CNS and peripheral tissues during development and adulthood, which is lacking at present. To address this, we employed zebrafish as a model. This choice reflects the outstanding qualities of the zebrafish as an *in vivo* model for studying vertebrate development [19], the documented presence of F-spondin in this species [12], and its striking capacity for active tissue renewal and regeneration throughout life [20]. Thus, the zebrafish provides an excellent opportunity to investigate the role of F-spondin in dynamic CNS and peripheral tissue modifications throughout the life-span of a vertebrate animal.

## Results

### Expression of the F-spondin Homologs in Zebrafish

**Figure 1:**
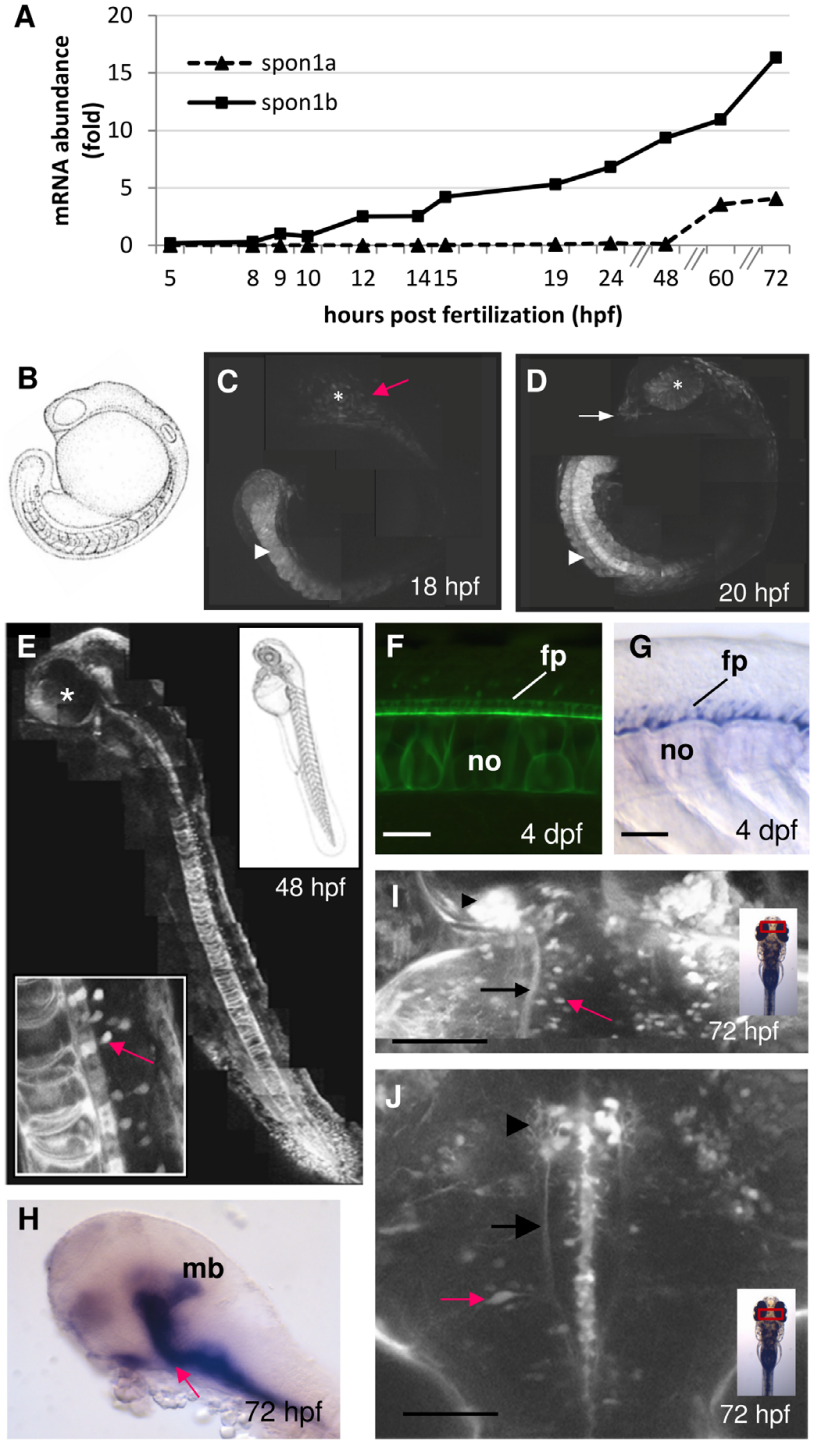
F-spondin expression during early zebrafish development. A. Onset of *spon1a* and *spon1b* expression during embryogenesis. Representative experiment showing mRNA abundance based on real-time RT-PCR (qPCR), with 1 fold corresponding to *spon1b* at 9 hpf. Zebrafish eggs fertilized at the same time (n>360) were sampled at intervals over a 72 h period (n = 30 embryos/larva per time point) and mRNA abundance for both genes was quantified in each same. **B.** Embryo schematic, 18 hpf. **C-D.** *Spon1b:GFP* expression in the tail bud, notochord and myotomes (arrowhead), brain areas (red arrow), olfactory bulbs (OB, white arrow) and retina (asterisk) at 18 hpf (B) and 20 hpf (D). Photomontages of confocal images. **E.** *Spon1b:GFP* expression pattern at 48 hpf. Photomontage of confocal images, sagittal view, asterisk: eye. Inset: developing motor neurons of the spinal cord (red arrow), dorsal to floor plate and notochord. **F-G.** The floorplate and notochord highlighted by *spon1b:GFP* (F, live image, 4 dpf) and *in situ* hybridization for *spon1b* mRNA (G, 4 dfp). Notochord (no), floorplate (fp). **H.** *Spon1b* expression in the flexural organ (arrow), and midbrain (mb). (*in situ* hybridization, 4 dpf). Rostral end to the left. **I.** Dorsal view of the telencephalon (Tel) and TeO border at 3 dpf, showing *spon1b* expression in habenula (arrowhead), in the fasciculus retroflexus (FR) emerging from it (black arrow), and in individual cells of the TeO (red arrow). Confocal z-stack image. **J.** Dorsal view of midbrain-hindbrain area at 3 dpf showing *spon1b:GFP* in the developing nMLF (arrowhead), in MLF projections (arrow) and in motor neurons of the reticular formation, including Mauthner cells (red arrow). Rostral end is up in I-J. Confocal z-stack image. Scale bars: F-G: 25 µm; I-J: 100 µm.

The two F-spondin homologs in zebrafish, Spon1a and Spon1b have an identity of 73% and 70%, respectively, with the human SPON1 protein. The identity between the two zebrafish homologs is 74%. Using real time quantitative RT-PCR (qPCR), the mRNA abundance for the two F-spondin homologs *spon1a* and *spon1b* was measured in developing embryos. The onset of *spon1b* mRNA expression occurred at 9–10 hours post fertilization (hpf), increasing 6.8 fold by the end of the first day of development, and reaching 16.3 fold by 72 hpf (Fig. 1A). In contrast, *spon1a* mRNA expression was initiated only after 48 hpf, and increased 4.0 fold by 72 hpf. Similarly, in adult zebrafish, mRNA abundance for *spon1b* was 2.0 fold and 72.3 fold higher than for *spon1a* assessed in the same brain and eye tissue samples, respectively (n = 3–4 fish; for brain, paired t(3) = 2.90, p<0.03; for eye, paired t(2) = 10.06, p<0.0005. The higher abundance of *spon1b* mRNA in larval and adult zebrafish suggested a potentially more important role for this homolog. We, thus, proceeded to characterize the expression patterns for *spon1b* in developing and adult zebrafish using three complementary approaches. These included the imaging of a fluorescent transgene signal (*in vivo* or in fresh-frozen brain tissue), immunohistochemical (IHC) staining for GFP, and *in situ* hybridization (ISH) to localize *spon1b* mRNA expression. The first two techniques allowed for visualizing both the F-spondin positive cells and their projections, while ISH highlighted the cell bodies only. The results were typically consistent among the methods used and are referred to below as *spon1b* or F-spondin expression, with any inconsistencies between methods discussed, where applicable.

### The Transgenic *spon1b:GFP* Zebrafish Reveals *in vivo* Patterns of F-spondin Expression During Vertebrate Development

To study the anatomical distribution of *spon1b* expression throughout zebrafish development and maturation, we used the 10.3 kb upstream promoter region of the *spon1b* gene (Accession# NM_131517) to drive expression of a fluorescent marker, the enhanced green ffiuorescent protein (EGFP, also referred to here as GFP). This construct was used to establish a stable *spon1b*:GFP transgenic zebrafish line. In *Tg(spon1b:GFP)* fish, GFP expression was first detected around 15–16 hpf, being visible along the developing body axis, with a stronger signal in the head and tail regions (data not shown). This is consistent with the onset of *spon1b* mRNA production at 10 hpf, as per qPCR, and allowing several hours for the accumulation of newly-synthesized GFP. Thereafter, rapid increases in *spon1b:GFP* expression were documented in the embryonic tail bud, notochord and myotomes (Fig. 1B-D). By 18 hpf, the *spon1b:GFP* signal was clearly visible in the brain and eye regions, with robust expression in the developing retina and olfactory bulb by 20 hpf (Fig. 1C-D). During this period, F-spondin expression intensified along the developing notochord, and strongly labeled the entire row of individual cells of the floor plate (Fig. 1E-G). Although both the transgene and ISH revealed this F-spondin localization during development, the expression in the notochord was more robust *in vivo*, in the *Tg(spon1b:GFP)* fish (Fig. 1F-H). The *spon1b:GFP* signal in the most anterior end of the notochord, known as the flexural organ, was particularly pronounced (Fig. 1H). This region has been previously identified as a source of Reissner’s fiber-related proteins, including F-spondin [21]. As development proceeded, a gradual increase in F-spondin expression along the developing spinal cord was associated with the early developing Rohon-Beard sensory neurons, and with the development and extension of primary motor neurons (Fig. 1E, inset). The axons of the latter were strongly labeled in the *Tg(spon1b:GFP)* zebrafish, highlighting the descending pathways. These are known to innervate restricted domains within each myotome and contribute to the first muscular contractions initiated around 17 hpf [22]. The developing midbrain and diencephalic regions were rich in F-spondin starting at 24 hpf, and this could be observed *in vivo* in *Tg(spon1b:GFP)*, and using whole mount *in situ* hybridization (Fig. 1H-J). By 72 hpf, an especially strong *spon1b:GFP* signal was localized to the neurons of the laterally-positioned habenula (Hb) nulclei and their descending projections forming the fasciculus retroflexus (FR; Fig. 1I). In the midbrain, F-spondin positive cells were documented in the optic tectum (TeO) and in the nucleus of the medial longitudinal fasciculus (nMLF) (Fig. 1I-J). The distinct motor neurons of the hindbrain were also rich in F-spondin expression, including the large paired Mauthner cells (Fig. 1J).

### F-spondin Expression in Adult Zebrafish Brain

#### Telencephalon

**Figure 2:**
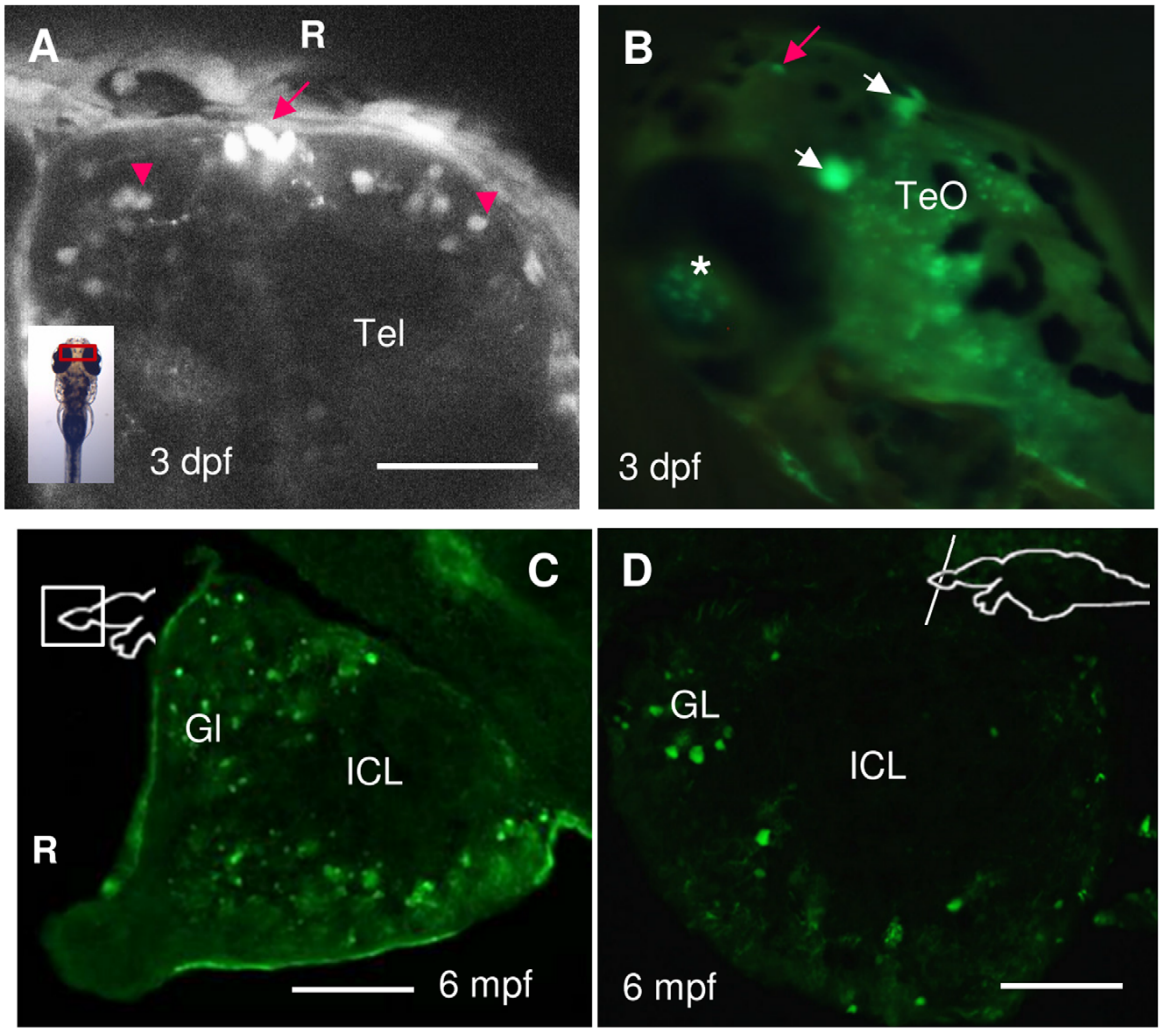
Expression of *spon1b:GFP* in the olfactory bulbs (OB) and telencephalon (Tel) of larval and adult zebrafish. **A.** Dorsal Tel of 3 dpf embryo, showing individual cells of the OB (red arrowheads), and a cluster of three oval midline cells at the OB-Tel boundary (red arrow). Confocal z-stack image. **B.** Latero-dorsal view of live larva, at 3 dpf, with robust *spon1b:GFP* expression in the oval cells of the Tel-OB boundary (red arrow), in the paired habenular nuclei (white arrows), in individual cells of the TeO, and in the eye, with retinal ganglion cells visible through the lens (asterisk). **C-D.** Sagittal (B) and coronal (C) sections of the adult OB showing *spon1b*-positive cells in the outer glomerular cell layers (GL), but not in the inner cell layers (ICL). R: rostral end, in A & C. Scale bars: A: 200 µm; C-D: 50 µm.

**Figure 3:**
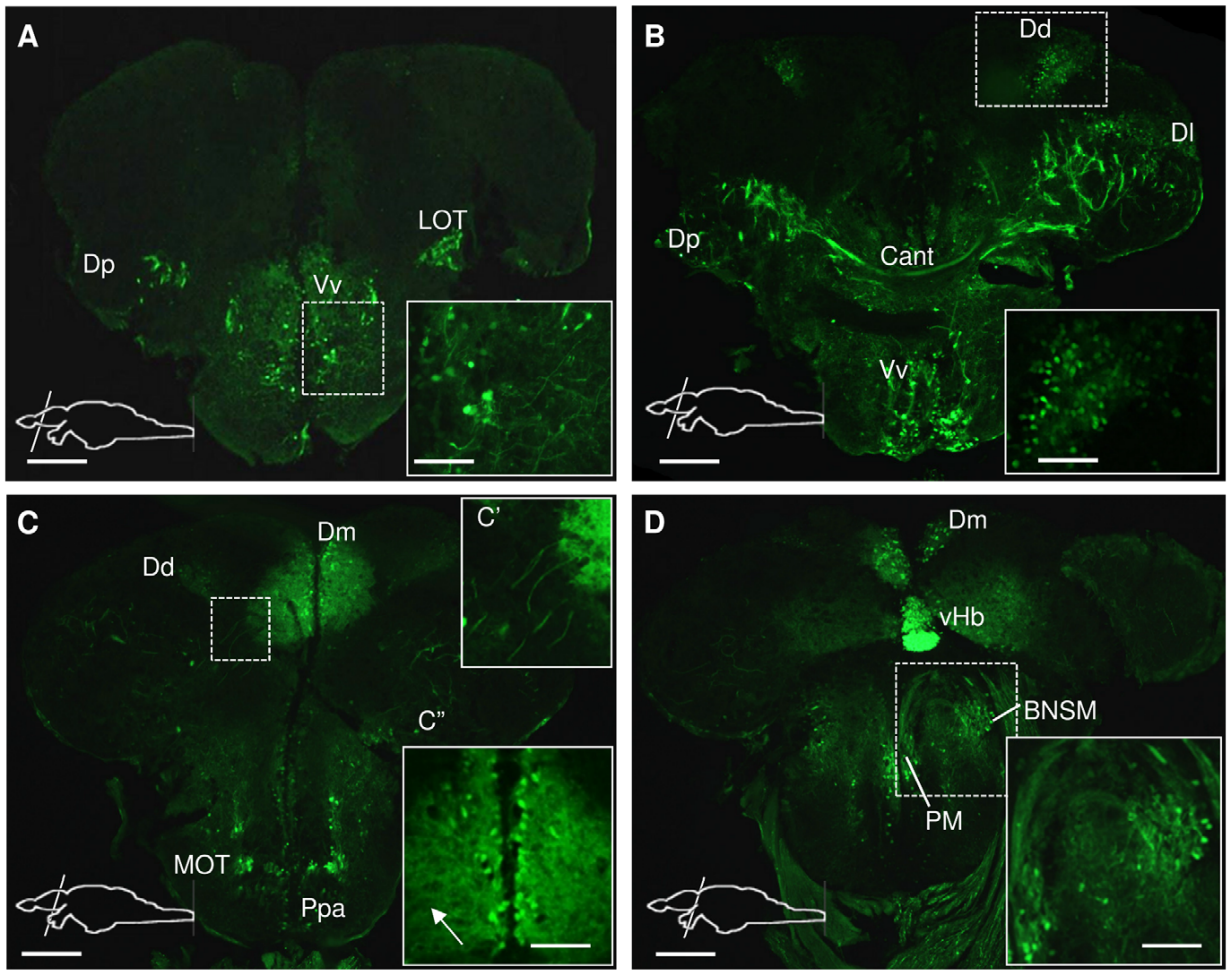
*Spon1b* expression in the telencephalon of adult zebrafish. Immunostaining for GFP in *Tg(spon1b:GFP),* coronal sections from rostral (A) to caudal (D) end. **A.** *Spon1b* expression in the central nucleus of ventral Tel (Vv), and terminal projections of the lateral olfactory tract (LOT), medial to dorsal nucleus of dorsal Tel (Dp). Inset: thick projections of *spon1b*-positive cells in Vv. **B.** *Spon1b* expression in Vv and the lateral (Dl), posterior (Dp) and dorsal (Dd) nuclei of the dosal Tel. Inset: small positive cells in Dd surrounding sulcus ypsiloniformis. Cant: anterior commissure. **C.** Robust *spon1b* expression in the medial nucleus of the dorsal Tel (Dm) and in the medial olfactory tract (MOT). Inset C’: Thick and long projections, originating from LOT area. Inset C”: medially located *spon1b* positive cells in Dm with thin and dense projections extending laterally throughout the nucleus (arrow). Ppa: parvocellular preoptic area **D.** *Spon1b* expression in the preoptic area. Inset: magnocellular nucleus (PM) and bed nucleus of stria medullaris (BNSM). Strong *spon1b* expression in the ventral habenula (vHb) (only the right vHb is visible). Scale bars: A-D: 200 µm. Insets: 50 µm.

The robust *spon1b* expression in the olfactory area and telencephalon (Tel, Fig. 1D, 2A-D), that was apparent at 20 hpf in the zebrafish embryo, remained prominent thereafter. In the adult olfactory bulbs (OB), *spon1b*-positive cells and thick projections were distributed within the glomerular (GL) cell layer, but not the internal cell layer (ICL) (Fig. 2B-C). The medial and lateral olfactory tracts (LOT/MOT), originating from these OB layers, could be traced throughout the ventral and dorsal Tel (Fig. 3A-B). A midline cluster of three large oval *spon1b*-positive cells was consistently present at the boundary of the OB and Tel in both larval and adult zebrafish (Fig. 2A,D red arrows). The origin of these distinct cells is unknown to us.

In the telencephalon (Tel), the *spon1b* transgene signal and ISH revealed several F-spondin-positive regions. In the periventricular area of the ventral telencephalon (subpallium), *spon1b* was expressed in the ventral nucleus (Vv), which is suggested to be the teleost homolog of the mammalian septal nuclei [23], [24]. The entire rostro-caudal extent of the Vv was immunopositive for *spon1b:GFP*, with labeled cells spanning the region from the medial periventricular zone to the most lateral boundary of the nucleus (Fig. 3A). The densely labeled LOT projections terminated in the posterior nucleus of the dorsal Tel (Dp), the primary olfactory area in zebrafish [23]. Their *spon1b*-positive fibers also traveled toward the medial nucleus of the dorsal Tel (Dm) and partially crossed into the contralateral hemisphere via the anterior commissure (Cant, Fig. 3B-C).

In the dorsal pallium (D), *spon1b* was present in the cells of the medial nucleus, Dm (Fig. 3C-D), a likely homolog of the basolateral amygdala in mammals [25], [26]. The *spon1b*-positive cells were located in the midline region of Dm, periventricularly, with their dense projections being constrained to the boundaries of the nucleus (Fig. 3B-C). A few weakly stained cells could be observed in the lateral (Dl) and posterior (Dp) nuclei of D (Fig. 3B-D). *Spon1b*-positive cells were observed in the dorsal nucleus of D (Dd), surrounding the sulcus ypsiloniformis (Fig. 3B-C). This nucleus is considered to be part of the processing center for somatosensory information from the lateral line [23].

In the preoptic region, *spon1b* expression was strong and abundant in the anterior parvocellular nucleus (Ppa; Fig. 3C). Individual cells and their dorsolaterally projecting axons were evident in the most lateral regions of the Ppa. More caudally, the magnocellular preoptic nucleus (PM) and its dorsal projections were strongly immunoreactive, while fewer positive cells were present in the medial region of the posterior parvocellular nucleus (PPp, Fig. 3D). The most posterior parts of the Ppp, and the suprachismatic nucleus appeared to be free of *spon1b* expressing cells (Fig. 3D). More laterally, *spon1b* was expressed in the bed nucleus of the stria medullaris (BNSM, Fig. 3D), a recently described nucleus in adult zebrafish [27].

#### Diencephalon

**Figure 4:**
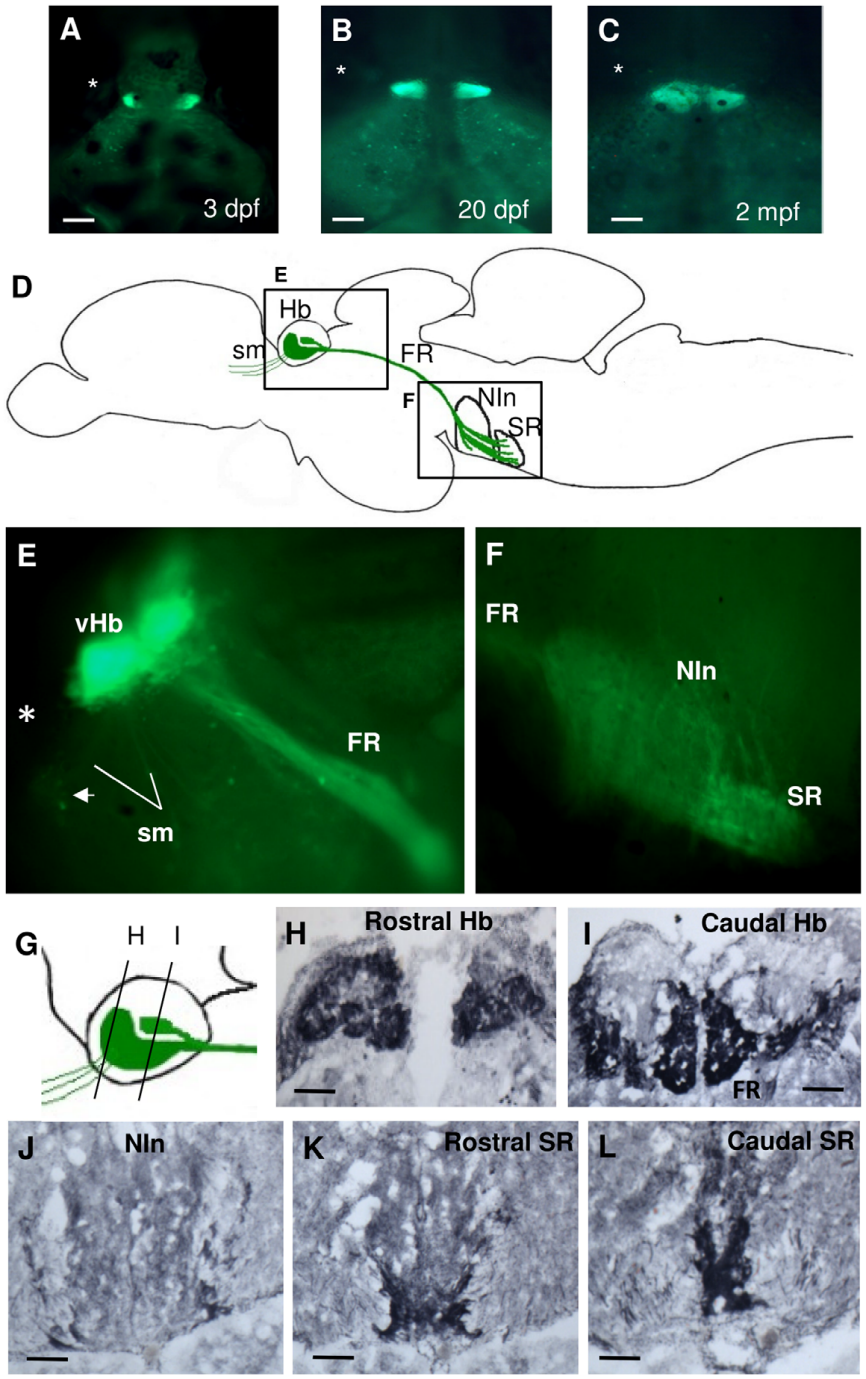
*Spon1b:GFP* expression in the habenular nuclei and their projections. **A-C.** Dorsal view of live zebrafish showing migration of ventral habenular nuclei from lateral to medial position: 3 dpf (B), 20 dpf (C), and 2 mpf (D). Asterisk indicates the position of the eye. **D.** Schematic of DCP in adult zebrafish showing areas of *spon1b:GFP* expression (E and F) and depicting the habenula (Hb), its afferent projections within the stria medullaris (sm), its efferent projection: fasciculus retroflexus (FR), and target nuclei: interpeduncular nucleus (NIn), and superior raphe (SR). **E-F.** Para-sagittal cut through fresh-frozen adult Tg(*spon1b*-GFP) brain. GFP fluorescence highlights all DCP structures, including vHb nuclei, FR and sm (E). Also, cells of the bed nucleus of the stria medullaris (BNSM, arrow in E), and projections to NIn and SR in F. Rostral to the left. **G.** Schematics of the relative shape and position of the *spon1b*-positive nuclei of the Hb, as shown in H-I. **H-L**. Coronal sections immunostained for GFP: rostral (H) and caudal (I) Hb, NIn (J), and rostral (K) and caudal (L) SR. Scale bars: A-B: 100 µm; D: 25 µm; H-L: 50 µm.

Pronounced spon1b:GFP expression was documented in the epithalamus and Dorsal Conduction Pathway. This was especially robust in the habenula (Hb), which displayed a strong fluorescence signal in the developing and adult Tg(*spon1b:GFP*) fish (Fig. 2A and 4A-I). In larvae, the transgene was expressed only in the laterally-positioned Hb nuclei, located at the dorsal surface of the developing brain (Fig. 1I, 4A). As development proceeded, the *spon1b:GFP* positive Hb nuclei increased in size and gradually moved medially (Fig. 4A-C). By 3 months post fertilization (mpf) the Hb nuclei were adjacent to each other at the midline (Fig. 4C). In both developing and adult *Tg(spon1b:GFP)* zebrafish, the entire dorsal conduction pathway (DCP) was highlighted, visualizing the fasciculus retroflexus (FR), projecting from the Hb to the interpeduncular nucleus (NIn) and superior raphe (SR) (Fig. 1I, 4D-L).

**Figure 5:**
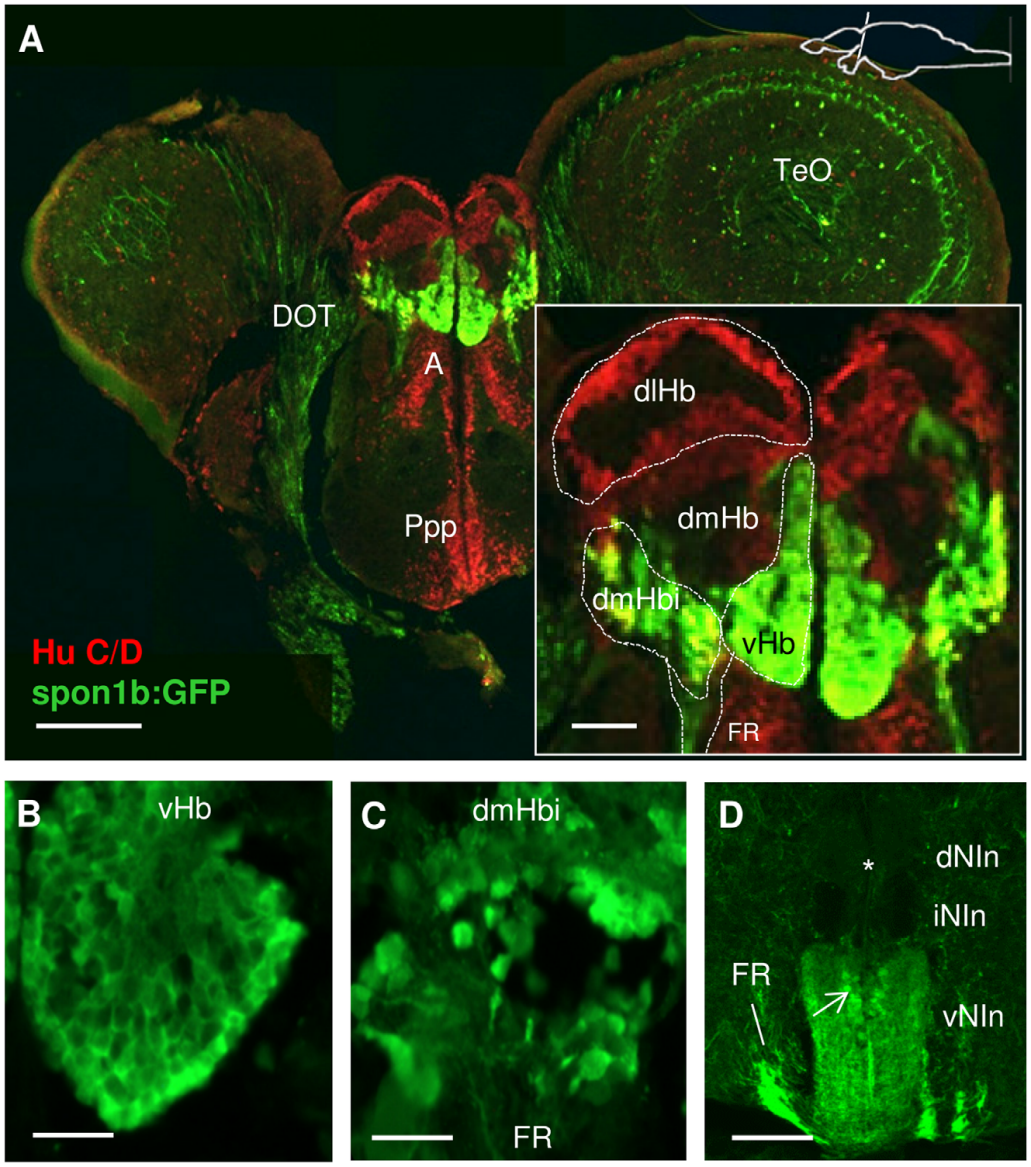
Expression of *spon1b* and Hu C/D in the habenula (Hb) complex. **A.** Coronal section through the Hb showing *spon1b:GFP* immunoreactivity in green and the pan-neuronal marker Hu C/D in red. Note *spon1b* negative areas at this level: Anterior nucleus of thalamus (A), posterior preoptic areas (ppp). Inset to (A): schematic subdivisions of the Hb in *spon1b*-positive ventral nucleus (vHb), inferior nucleus of dorsomedial zone (dmHbi), and *spon1b*-negative dorsolateral (dlHb) and dorsomedial (dmHb) zones. **B-C.** High magnification images of the small densely packed cells in vHb (B) and the larger sparse cells in dmHbi (C). **D.** Coronal view of the interpeduncular nucleus (NIn) showing *spon1b-*positive terminal projections to the ventral area (vNIn), but not the dorsal and intermediate Nin (dNIn, iNIn) (asterisk). Note bypassing fibers from FR circumventing NIn on its way to the SR, and *spon1b-*positive cells at the core of Nin (arrow). Scale bars: A: 200 µm, inset: 50 µm; B-C: 25 µm; D: 100 µm.

Consistent with the earlier findings that the laterally-positioned Hb nuclei in larvae migrate ventrally during maturation [28], the strongest *spon1b* labeling was present in the ventral Hb (vHb) in adult zebrafish (Fig. 4G-I, 5A). The paired symmetrical vHb nuclei each have a conical shape defined by a wide rostral end, occupying the entire anterior region of the Hb, and a narrow caudal end (Fig. 4H-I). Moreover, a region located dorsolateral to the vHb, and separated from it by the emerging FR, was also *spon1b:GFP* positive (Fig. 4I, 5A). Based on the localization of this additional area, we have named it the inferior subnucleus (dmHbi) of the earlier described dorsomedial nucleus of the Hb (dmHb) [29], [30]. In contrast to the vHb having small densely packed cells (Fig. 5B), dmHbi had relatively large and sparsely positioned cells (Fig. 5C). Both of these cell types double-stained for *spon1b:GFP* and Hu C/D, indicating their neuronal origin (Fig. 5A). It should be noted that the vHb cells stained more weakly for Hu C/D than those in the adjacent dmHbi nucleus. The rest of the dorsal Hb was also immunopositive for Hu C/D, but not for *spon1b:GFP* (Fig. 5A).

Differentiating between the two *spon1b:GFP* positive areas of Hb was further assisted by the distinctly different targets of dorsal and ventral Hb nuclei. In zebrafish, the vHb projects to the raphe nuclei [28]. In contrast, the two principal nuclei of dorsal Hb have asymmetric projections. The dorsolateral Hb (dlHb) projects to the dorsal part of the interpeduncular nucleus (dNIn), while fibers originating in the dmHb are traced to ventral and intermediate NIn (vNIn, iNIn) [29], [30]. Accordingly, *the spon1b*-positive FR fibers were present in ventral but not the dorsal NIn, consistent with the dmHb pathway (Fig. 5D), and circumvented the NIn on their way to the SR (Fig. 4J-L, 5D), consistent with the vHB projections [28]. These latter fibers were seen as densely packed *spon1b-*positive terminals in ventral regions of SR (Fig. 4F, K-L). *Spon1b* expression was present in cells of the NIn core but not in the SR (Fig. 4J-L). Moreover, projections between the Hb and BNSM, as well as some ventral projections, were seen in sagittal brain sections (Fig. 4E). Considering the conserved connectivity of the dorsal conduction pathway in vertebrates [31], these projections were, presumably, part of the stria medullaris.

Unlike in the vast majority of brain areas where ISH for *spon1b* recapitulated *spon1b:GFP* expression, the *spon1b* mRNA signal was detected in additional nuclei of the dorsal Hb (data not shown) that were not revealed by the transgene. This discrepancy is unlikely to result from a positional effect of the transgene integration site, since our two independent transgenic founder lines had similar patterns of Hb expression, highlighting only laterally-positioned Hb nuclei in larvae. Potentially, this result could indicate that additional promoter or enhancer regions, beyond the 10.3 kb upstream fragment, are required for the expression of *spon1b* in dorsal Hb nuclei.

#### Thalamus

**Figure 6:**
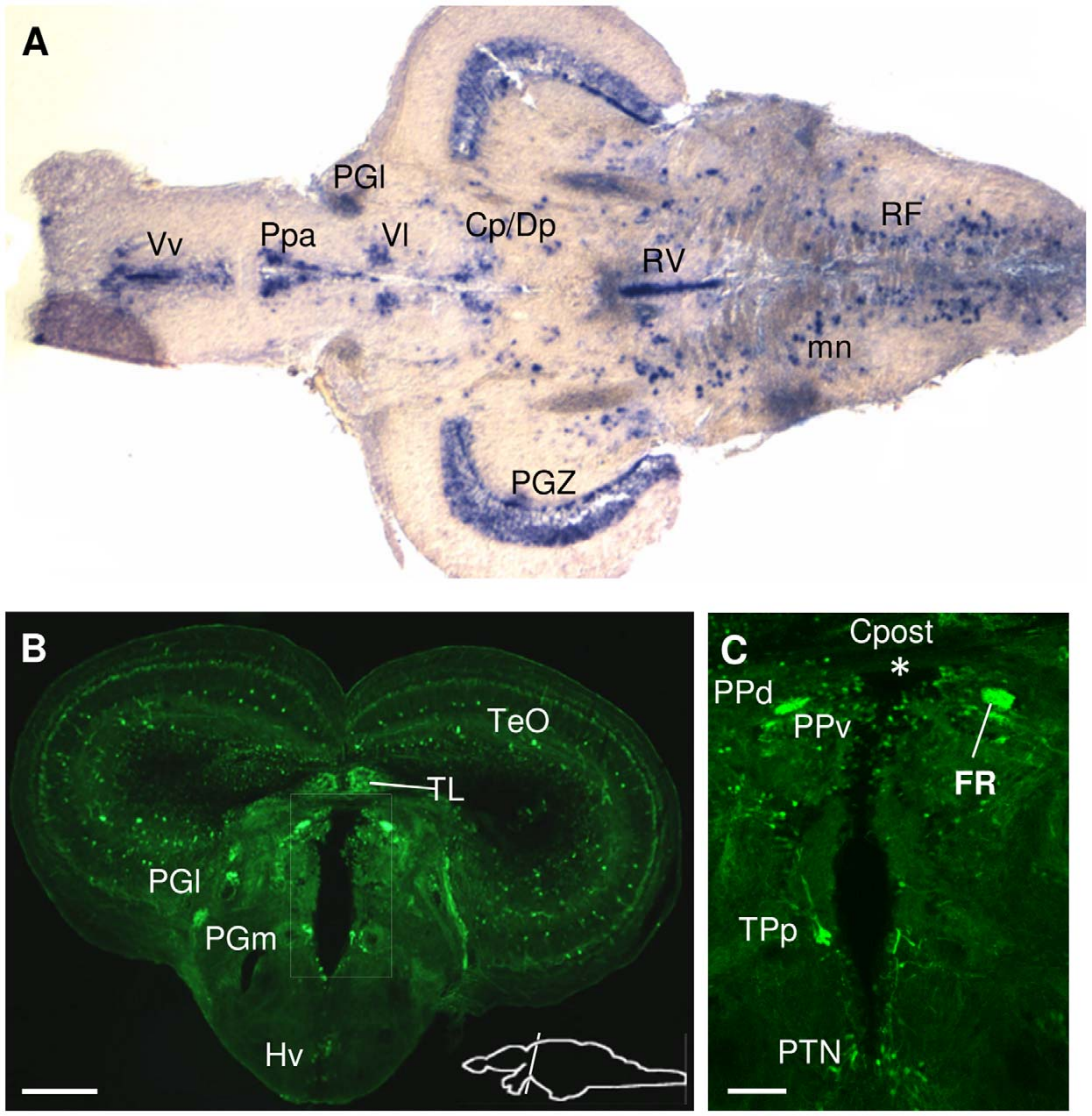
*Spon1b* expression in thalamic and pretectal regions. A. Horizontal section showing *spon1b* mRNA-positive nuclei (in situ hybridization): dorsoposterior (DP), centroposterior (CP) and ventrolateral (Vl) thalamic nuclei; anterior parvocellular preoptic (Ppa); Ventral Tel (Vv). **B.** Coronal section through the thalamus, immunostained for GFP. Medial (PGm) and lateral (PGl) preglomerular nuclei, torus longitudinalis (TL), ventral hypothalamus (Hv), optic tectum (TeO). **C.** Periventricular region (boxed area in B in adjacent section), showing *spon1b*-positive cells in the dorsal (PPd) and ventral (PPv) periventricular pretectal nuclei, in the posterior tuberculum (TPp), in the posterior tuberal nucleus (PTN) and in fibers of the fasciculus retroflexus (FR). Note absence of signal in the subcommissural organ (asterisk: SCO region, ventral to posterior commissure, Cpost). Scale bars: A-B: 100 µm; C: 50 µm.

**Figure 7:**
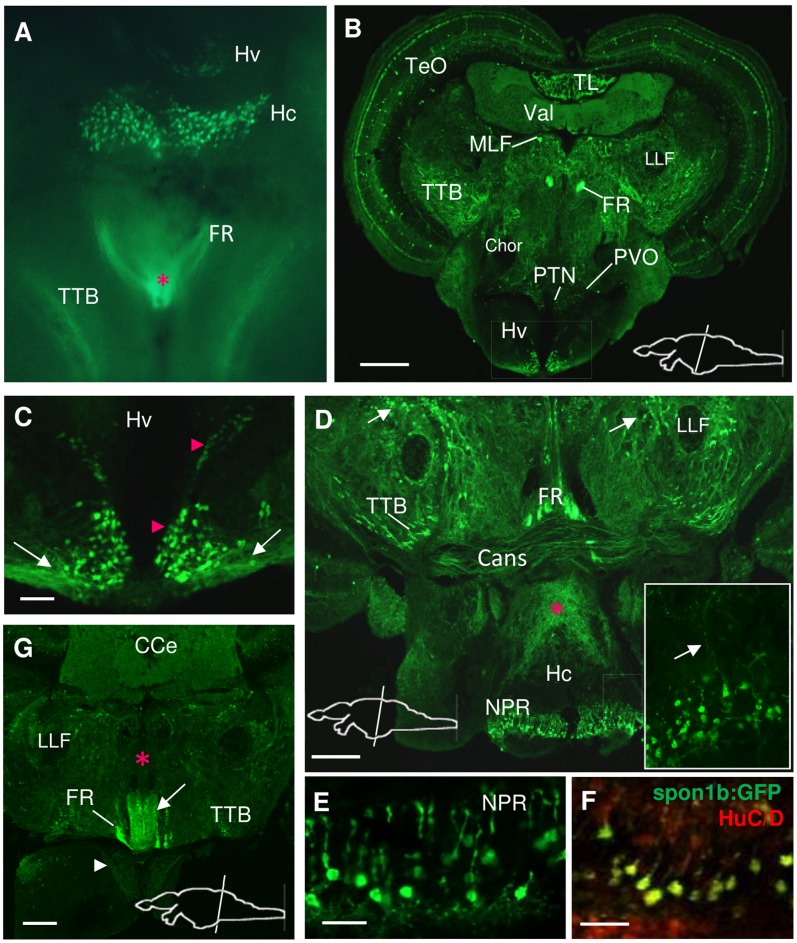
*Spon1b* expression in the hypothalamic area. **A.** Ventral view of a freshly-dissected adult Tg(*spon1b-GFP*) brain: fluorescence signal in the ventral (Hv) and caudal (Hc) hypothalamic nuclei, the tectobulbar tract (TTB), the interpeduncular nucleus and superior raphe (asterisk), and fibers from the fasciculus retroflexus (FR). **B.** Coronal section at the level of the ventral hypothalamus (Hv), immunostained for GFP. Note the strong fluorescence signal in FR and medial longitudinal fasciculus (MLF), but its absence in the horizontal commissure (Chor) and lateral lemniscus (LLF). Optic tectum (TeO), posterior tuberal nucleus (PTN), paraventricular nucleus (PVO), torus longitudinalis (TL), valvula cerebelli (Val). **C.** *Spon1b* expression in periventricular cells (arrowheads) of the Hv nucleus (boxed area in B) with long projections extending laterally (arrows). **D.** Coronal section at the level of the caudal hypothalamus (Hc) showing high *spon1b* expression in the nucleus of the posterior recess (NPR), and long projections within the dorsal hypothalamus (asterisk). These projections, in part, originate from the most lateral cells of the NPR (arrow in inset). Note strong signal in scattered cells and projections in the tegmentum (arrows), ansulate commissure (Cans), and tectobulbar tract (TTB). **E.** Robust *spon1b* expression in the NPR. Note the dorsal projections of CSF-contacting neurons. **F.** Cells in NPR showing co-localization of the pan-neuronal marker Hu C/D (red) and *spon1b:GFP* (green). **G.** Coronal section at the level of NIn showing *spon1b:GFP* in vNIn (white arrow), but its absence in dorsal NIn (dNIn, red asterisk). *Spon1b*-positive fibers in the cerebellar corpus (CCe), FR (fibers circumventing NIn on the way to SR), mammillary bodies (arrowhead) and TTB. Scale bars B,G: 100 µm; C: 25 µm, D: 50 µm; E-F: 20 µm.

The ventrolateral thalamic nucleus, which is a relay station for visual and other sensory inputs to the optic tectum (TeO) [32], contained large, widely distributed cells with strong *spon1b:GFP* expression (Fig. 6A). The central and dorsal posterior thalamic nuclei (CP/DP), the medial and lateral preglomerular nuclei (PGm, PGl), the paraventricular organ (PVO) and the posterior tuberal nucleus (PTN) contained *spon1b* expressing cells (Fig. 6–7). Large *spon1b*-positive CSF-contacting cells of the posterior tuberculum (TPp) extended their neurites laterally, while numerous thinner projections were visible throughout the parenchyma of the thalamus, especially in its anterior region (Fig. 6C).

#### Hypothalamus

Strong *spon1b* expression was revealed in two distinct periventricular areas of the rostral and caudal hypothalamus (H, Fig. 7). Their extreme ventral location allowed for the observation of the *spon1b:GFP* positive cells on the ventral side of the whole brain dissection, using fluorescence microscopy (Fig. 7A). The rostral area, corresponding to the ventral nucleus of the hypothalamus (Hv) that surrounds the mediobasal region of the diencephalic ventricle, contained a well-defined population of *spon1b*-positive cells. These extended their projections laterally, forming a distinct tract, coursing along the ventral surface of the hypothalamus (Fig. 7B-C).

In the inferior lobe, in the caudal zone of the periventricular hypothalamus (Hc), a distinct horizontal band of the nucleus of the posterior recess (NPR) [32] displayed large and densely packed *spon1b*-positive cells, positioned along the ventral wall of the diencephalic ventricle (Fig. 7D-F). These CSF-contacting neurons extended their prominent processes dorsally, toward the lumen of the posterior recess of the diencephalic ventricle and stained for Hu C/D, confirming their neuronal nature (Fig. 7E-F). A dense network of *spon1b* positive fibers could be observed in the dorsal region of the Hc, extending dorsomedially toward the tegmental areas (Fig. 7D). Part of those projections could be seen originating from the cells in the caudal and lateral aspects of the posterior recess of the DiV (Fig. 7D, inset). A weak *spon1b* signal from scattered thin projections was documented in the mammillary bodies (Fig. 7G).

#### Pretectum

In larval zebrafish, strong *spon1b* expression was present in the nucleus of the medial longitudinal fasciculus (nMLF), with *spon1b:GFP* highlighting its long projections along the ventral border of the rhomboencephalic ventricle and ventral region of the spinal cord (Fig. 1J). The distinct cells of this nucleus and its projections, which communicate multimodal sensory information and locomotor commands in zebrafish [33] continued to express *spon1b* in adults (Fig. 7B). Both periventricular pretectal nuclei, dorsal and ventral (PPv, PPd), contained small-size cells with strong *spon1b* expression (Fig. 6C).

#### Mesencephalon

**Figure 8:**
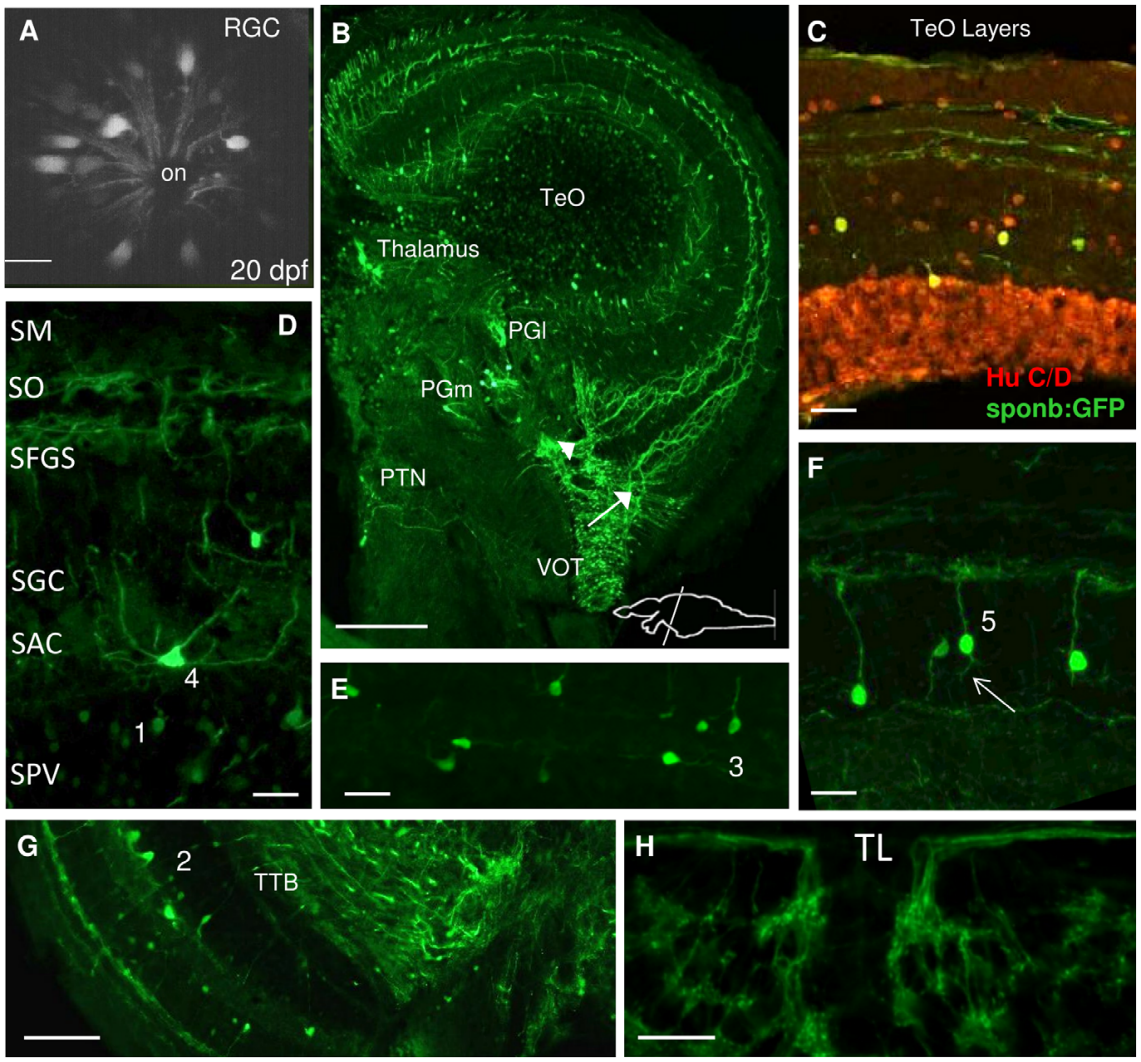
*Spon1b* expression in areas of the visual system. **A.** *Spon1b* expression in retinal ganglion cells (RGC) forming optic nerve (on) in live zebrafish at 20 dpf. Confocal image. **B.** Coronal section of adult zebrafish fish brain at the level of the optic tectum (TeO) showing *spon1b-*positive retino-tectal projections of the ventrolateral optic tract (VOT) splitting into two TeO layers: *stratum fibrosum et griseum superficiale* (SFGS, arrow) and *stratum opticum* (SO, arrowhead). *Spon1b*-positive cells in the medial (PGm) and lateral (PGl) preglomerular nuclei, and in the posterior tuberal nucleus (PTN). **C.** Double immunostaining in TeO demonstrates co-localization of *spon1b:GFP* (green) and Hu C/D (red) signal, confirming the neuronal nature of *spon1b*-positive cells. **D.** Coronal section identifying six TeO layers, with *spon1:GFP* expression in retinotectal projections in SO and SFGS, cell types 1 (in SPV border) and 4 (in SAC). **E.** Type 3 TeO cell, with horizontal projections in the SAC layer. **F.** Type 5 TeO cells with vertical projections in the SGC layer. **G.** Type 2 TeO cell with vertical projections traversing all TeO layers and forming the tectobulbar tract (TTB). **H**: *Spon1b*-positive cells and rami-like neuropil in the torus longitudinalis (TL). Scale bars: A: 10 µm; B: 100 µm; E-F: 15 µm; D,H: 20 µm, G: 50 µm.

Another region of the brain with pronounced staining for *spon1b* was the TeO (Fig. 8). The retinal ganglion cells (RGC) expressed *spon1b* during early development, and continued expressing it throughout zebrafish life (Fig. 8A). In adult brain, *spon1b*-positive retinotectal projections of the ventral optic tract (VOT), coursed through the optic tract and innervated two distinct layers of TeO (Fig. 8D, arrows). These corresponded to the superior sublayer of the *stratum fibrosum et griseum superficiale* (SFGS) and *stratum opticum* (SO), where 80% and 15% of RGC terminate, respectively [34].

Individual *spon1b*-expressing cells were observed in the embryonic TeO around 48 hpf (Fig. 1J, 2D). In adult fish, five different types of *spon1b*-positive neurons could be identified in the tectal layers, based on their morphology and Hu C/D staining (Fig. 8C-G). A large number of widely distributed rounded cells with thin neurites were observed in the *stratum periventriculare* (SPV, Fig. 8D). Also in the SPV, rhomboid-shaped neurons were located at the most rostral and lateral TeO regions, extending thin apical dendrites toward superficial layers. Their long basal axons crossed the SPV, forming the tectobulbar tract (TTB) that projected on both sides of the NIn toward the hindbrain and spinal cord (Fig. 7D, 8G). The morphology of these cells is consistent with the earlier described TeO neurons that project from the torus semicircularis in rainbow trout [35], and are similar to cells described as the type IVa/b and V in goldfish [36]. In the same area, numerous multipolar *spon1b* positive cells with larger perikarya demonstrated prominent dorsally oriented processes (Fig. 8D). At the boundary of SPV and *stratum album centrale* (SAC), a smaller population of *spon1b*-positive neurons extended their processes alongside the horizontal axonal bundles (Fig. 8E).

In the *stratum griseum centrale* (SGC), a large population of neurons extended their apical dendrites toward the TeO surface, with elaborate arborizations terminating in the proximal SFGS and SO layers (Fig. 8F). Most of these neurons were unipolar, though some had short basal neurites or long descending axons that crossed inner TeO layers and contributed to the TTB (Fig. 8G). The pear-like shape of these neurons was similar to a previously described population of cells that project to the torus longitudinalis (TL) of the rainbow trout [37]. This was consistent with the dense *spon1b*-positive projections observed in TL of zebrafish (Fig. 8H). Moreover, TL contained granular cells organized in individual rami, strongly labeled with *spon1b:GFP* (Fig. 6B, 7B, 8H). Some of their long axons projected dorsolaterally, toward the most superficial layer of the TeO, the *stratum marginalis* (SM), while the majority joined the tectal commissure (Ctec) or coursed along the inner layers (Fig. 7B, 8H).

In the tegmentum, prominent *spon1b*-positive projections could be observed in the ansulate commissure (Cans, Fig. 7D). Dorsal and caudal to the Cans, thick fibers of the FR and TTB could be seen projecting to the NIn/SR and hindbrain motor neurons, respectively (Fig. 7D,G). This area also contained numerous *spon1b*-positive projections and scattered cell bodies throughout the torus semicircularis and superior reticular formation (Fig. 7B-D).

#### Metencephalon

**Figure 9:**
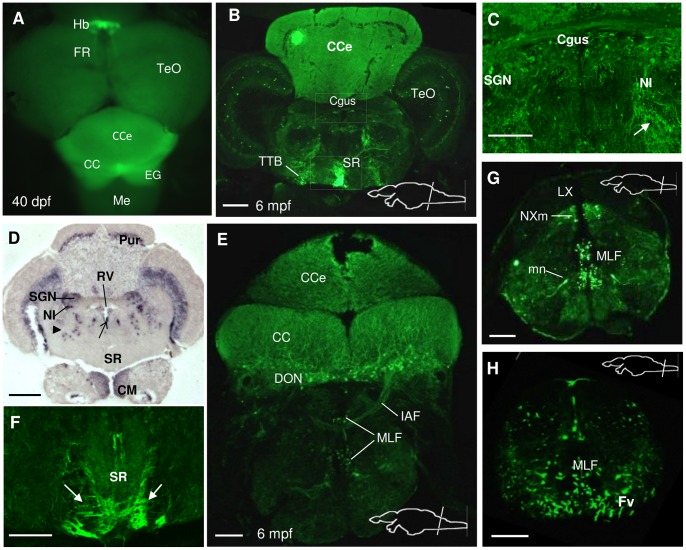
*Spon1b* expression in the hindbrain. A. Dorsal view of a freshly-dissected *Tg(spon1b:GFP)* juvenile zebrafish showing relative position of the paired habenular nuclei (Hb), emerging fasciculus retroflexus (FR), optic tectum (TeO), cerebellar corpus (Cce), granular eminence (EG), and medulla (Me). **B**. Coronal section of adult zebrafish brain at the level of the cerebellar corpus showing *spon1b:GFP* positive projections within the cerebellum, and to the superior raphe (SR). Tectobulbar tract (TTB). **C.** *Spon1b:GFP* signal in cells of the secondary gustatory nucleus (SGN), in the gustatory commissure (Cgus), and in scattered cells and projections throughout the tegmentum (arrow), and nucleus isthmi (NI). (High magnification picture of area in B). **D.** *Spon1b* mRNA expression (*in situ* hybridization) in Purkinje cell layer (Pur), ventral tip of rhomboencephalic ventricle (rv), SGN, nucleus isthmi (NI), superior reticular nucleus (arrowhead) and mammillary bodies (CM). Note the lack of signal in the Cgus and SR, confirming that the *spon1b:GFP* label in these regions comes from projections and not cell bodies. **E.** Coronal section at the level of the caudal cerebellum showing *spon1b*-positive cells and thick projections from cerebellar crest (CC), the descending octaval nuclei (DON) and the commissure that joins the nuclei, the internal arcuate fibers (IAF) coursing in between the axons of the medial longitudinal fasciculus (MLF). **F.** *Spon1b* **-**positive FR fibers (arrows) terminating on both sides of the ventral region of SR (high magnification image of area boxed in B). **G.** Coronal section at the level of the caudal medulla showing *spon1b:GFP* expression in the vagal motor nucleus (NXm), but not in the vagal lobe (LX). Note the symmetrically labeled motor neurons (mn), and the strongly labeled axons of the MLF and motor neuron axons in the ventral funiculus (Fv). **H.** Coronal section of the spinal cord showing *spon1b:GFP* expression in the axons of MLF and the ventral funiculus (Fv). Scale bars: B, F-H: 100 µm; C-D: 50 µm; E: 200 µm.

By 5 dpf, *spon1b* expression was visible at the most rostromedial region of the hindbrain. By 40 dpf, the *spon1b:GFP* signal could be observed in the projections within the *valvula cerebelli* (Val), cerebellar corpus (CCe), cerebellar crest (CC) and granular eminence (EG, Fig. 9A). In the adult brain, cells positive for *spon1b:GFP* were documented in the EG and CC of the cerebellum, and in the secondary gustatory nucleus (SGN), nucleus isthmi (NI), and superior reticular nucleus (SRN) (Fig. 9B-E), which are all derived from the upper rhombic lip (URL) and known to project to all lobes of the cerebellum [38]. Other *spon1b*-positive afferents to zebrafish cerebellum might have originated from the pretectal nuclei, torus longitudinalis, octavolateral region [38], which were also positive for the transgene. This wide range of *spon1b*-positive afferents explain, in part, the strong and uniform *spon1b:GFP* signal throughout the cerebellum. Large *spon1b*-positive cells were visible along both sides of the cerebellar midline, in the ventral anterior tip of the Val and around the dorsal surface of the Val and the CCe, marking the boundary between the molecular and granular cell layers (Fig. 9D). Their location and morphology are characteristic of Purkinje cells. It should be noted that these cells had a weak *spon1b:GFP* signal, though were well defined by *spon1b* mRNA *in situ* hybridization. While the *spon1b* mRNA signal was absent from the SR (Fig. 9D), GFP labeling was strong in *Tg(spon1b:GFP)* zebrafish (Fig. 9F), confirming that the signal comes not from cells but from axons originating in the Hb and traveling via the FR.

The most ventral tip of the rhomboencephalic ventricle (RV), medial to MLF, was strongly positive for *spon1b* (Fig. 6A, 9D). The *spon1b* mRNA expression in this area was especially well defined by ISH and extended throughout the entire RV to the level of the caudal medulla. This medullo-spinal region is characterized by the presence of CSF-contacting neurons, which are abundant in teleosts [39].

#### Myelencephalon

In the medulla oblongata, *spon1b* is expressed in multiple symmetrically positioned hindbrain neurons (Fig. 6A, 9E,G), including the large Mauthner cells (Fig. 1J), and in the neurons of the motor nucleus of X (Fig. 9G). In the spinal cord, the *spon1b*:GFP positive projections of these cells run along with the MLF and ventral funiculus (Fv, Fig. 9G-H). The tectobulbar tract (TTB) is also strongly labeled by *spon1b:GFP* (Fig. 7B-D, 8G, 9B), which sends motor output from the deeper layers of the TeO to the premotor reticulospinal system in the hindbrain [40].

### *Spon1b* is Expressed in Neurogenic Zones of the Zebrafish Brain

**Figure 10:**
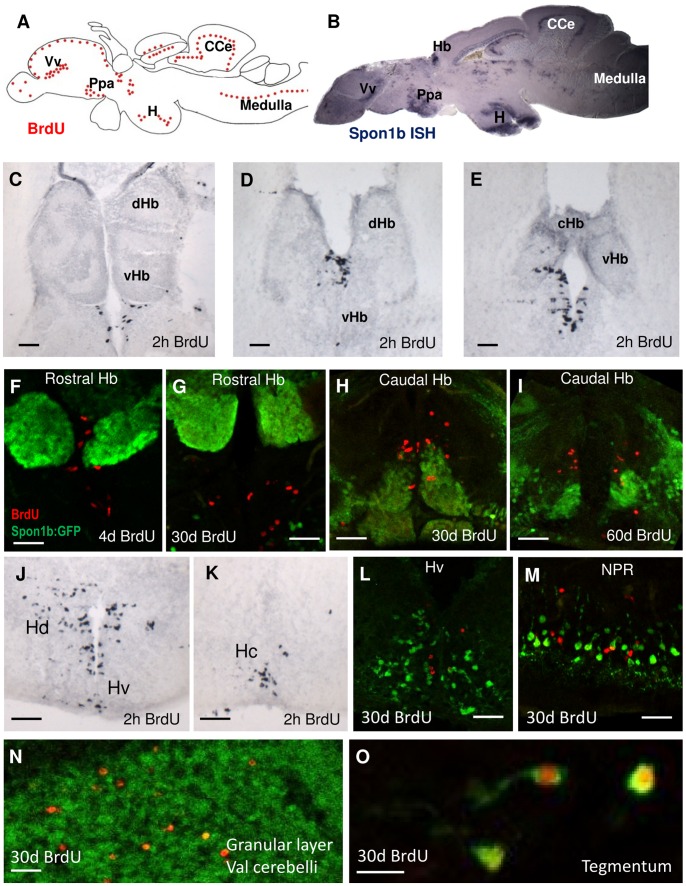
*Spon1b* is expressed in neurogenic niches. **A.** Schematic of neurogenic niches in zebrafish brain based on BrdU immunostaining and modified from Grandel *et. al.* (2006). Cerebellar corpus (CCe), Habenula (Hb), hypothalamic areas (H), parvocellular preoptic area (Ppa), thalamic area (T), ventral nucleus of ventral telencephalon (Vv). **B.** Mid-sagittal of adult zebrafish brain showing *spon1b* mRNA expression (*in situ* hybridization). Note the overlap of the *spon1b*-positive areas with the neurogenic niches. **C-E.** BrdU-positive nuclei in the habenular niche in rostral (D), and mid (E) and caudal (F) areas, 2 hours post-BrdU injection. **F-I.** Double immunostaining of the habenular niche, showing *spon1b*- (green) and BrdU- (red) positive cells, at 4d (F), 30d (G-H) and 60d (I) post BrdU-injection, showing migration of the BrdU nuclei away from the niche and into the surrounding tissue. **J-K.** BrdU nuclei in the ventral (J) and caudal (K) hypothalamus, Hv and Hc, respectively, 2 hours post-injection. **L-M.** Double immunostaining for BrdU (red) and *spon1b:GFP* (green) 30 days post BrdU injection in ventral hypothalamus (Hv, L), and in the nucleus of the posterior recess (NPR) in Hc (M) showing lack of co-localization. **N.** Double immunostaining for BrdU (red) and *spon1b:GFP* (green) 30 days post BrdU injection in the cerebellum. Note the BrdU-positive nuclei among the *spon1b*-positive fibers. **O.** Co-localization of BrdU (red) and *spon1b:GFP* (green) in cells extending long projections in the tegmentum 30 days post-BrdU injection. Scale bar C: 200 µm, D-L,N: 50 µm, M: 20 µm.

In zebrafish, adult neurogenesis is a continuous process, much more active than in mammals [20]. A total of 16 distinct proliferative niches have been identified in zebrafish brain. These are located mainly in periventricular areas, but also within the brain parenchyma [41]. We have documented a notable overlap of the areas of *spon1b* expression with proliferative zones in adult zebrafish brain (Fig. 10A-B). *Spon1b* expression is present within or next to BrdU-positive areas, such as the olfactory bulbs, ventral and dorsal telencephalon, parvocellular preoptic nuclei, Hb, ventral and dorsal thalamus, posterior tuberculum, posterior tuberal nucleus, hypothalamus, median optic tectum, torus longitudinalis, cerebellum, medulla and spinal cord (Fig. 10B). To understand the relationship between *spon1b* and stem cells, we examined cell proliferation patterns in several germinal zones in 1-year old zebrafish through a series of BrdU incorporation experiments. We examined brain tissue at 2 hours, 4 days, 1 month and 2 months after BrdU injection, documented *spon1b-* and BrdU-positive cell locations using double-immunohistochemistry, and searched for cells with co-localized *spon1b* and BrdU signals.

In the habenular neurogenic niche, the majority of BrdU-positive cells were located along the midline, adjacent to the diencephalic ventricle and vHb. In some specimens, the Hb niche extended ventrally, merging with the ventral thalamic niche. We observed a distinct rostro-caudal organization of the Hb niche, 2 hours post-BrdU injection. At the most rostral levels, the BrdU-positive cell nuclei were found immediately ventral to the vHb (Fig. 10C). More caudally, the dividing cells were concentrated in the medio-dorsal region of the vHb (Fig. 10D). Thereafter, at the level of the habenular commmissure (cHb), BrdU-positive nuclei again were located medio-ventral to the vHb nuclei (Fig. 10E). At all levels, few BrdU cells could be observed within the parenchyma of the Hb at 2 hours post-injection (Fig. 10C-E).

Four days after BrdU incorporation, the newly-divided cells could be found close to the periphery of the Hb niche, migrating dorsally along the ventricular walls and into the ventral and dorsal Hb (Fig. 10F). At 1 month post-injection, the surviving BrdU-labeled cells could not be detected in the niche *per se*, but were localized in the ventral and dorsal Hb nuclei (Fig. 10G-H). Over a 2-month post-injection period, most of the BrdU-labeled cells moved into the dHb nuclei (Fig. 10I). Importantly, none of the newly formed cells that migrated into the vHb or dHb expressed *spon1b* at any post-injection interval examined.

The periventricular hypothalamic niche in zebrafish is divided into dorsal, ventral and caudal zones (Fig. 10J-K), corresponding to their location in specific periventricular hypothalamic regions (Hd, Hv and Hc, respectively [41]). *Spon1b*-positive cells were abundant in the Hd, Hv and Hc, being close to BrdU-positive cells but not co-localizing with them at 2 hours or 30 days post-BrdU administration (Fig. 10L-M). The *spon1b*-positive cells were positioned in the peri-ventricular regions and parenchyma of the hypothalamic nuclei, while the BrdU-positive cells were found along the ventricular wall (Fig. 10J-M), consistent with previous observations [41], [42]. One month after BrdU injection, some of the newly formed cells had migrated away from the ventricle and into the parenchyma, although the majority remained closely associated with the niche. Similar to those in the Hb niche, the BrdU-positive cells of the hypothalamus did not co-localize with *spon1b:GFP* positive cells one or two months after cell division had occurred (Fig. 10L-M).

The cerebellar niche is one of the largest and most complex proliferation zones of the teleost brain, and the site of origin of the majority of new cells in the zebrafish brain [43]. It is also the only niche in the zebrafish that is not associated with the ventricles [43], [44]. The BrdU positive nuclei were found throughout the molecular cell layer of the Val, CCe, CC and EG at 2 hours after injection, consistent with earlier findings [43]. At 1 and 2 months post injection, the new cells had migrated into the granular cell layer of the cerebellum and could be observed among the *spon1b:GFP* positive fibers (Fig. 10N). No cells with co-localized *spon1b* and BrdU signals were found in the cerebellum for up to 2 months post-BrdU injection.

Upon close examination, the only area of the brain where we found BrdU-positive cells co-localizing with *spon1b:GFP* was the tegmentum (Fig. 10O). One month after the division occurred, these new cells had extended relatively long projections, highlighted by a GFP signal, and their morphology was consistent with a neuronal phenotype. Most likely, these new cells migrated from the posterior mesencephalic lamina niche or from the dorsal tectal proliferation zone [41].

### Expression of *spon1b* in Peripheral Tissues

We have documented that F-spondin expression is not limited to CNS but also present in a number of peripheral tissues in developing and adult zebrafish. Unlike in the brain or eye of adult zebrafish, very low levels of *spon1b* mRNA abundance could be detected by qPCR in zebrafish muscles and skin (28.8 and 11.9 fold less than in zebrafish brain, respectively), with no apparent transgene expression found. Nevertheless, as in the CNS, in both of these tissues the abundance of *spon1b* mRNA was consistently higher than that of *spon1a* (n = 2−4; unequal variance t-test, muscle vs. brain t(2) = 9.22 p<0.005, skin vs. brain t(4) = 3.23 p<0.001).

**Figure 11:**
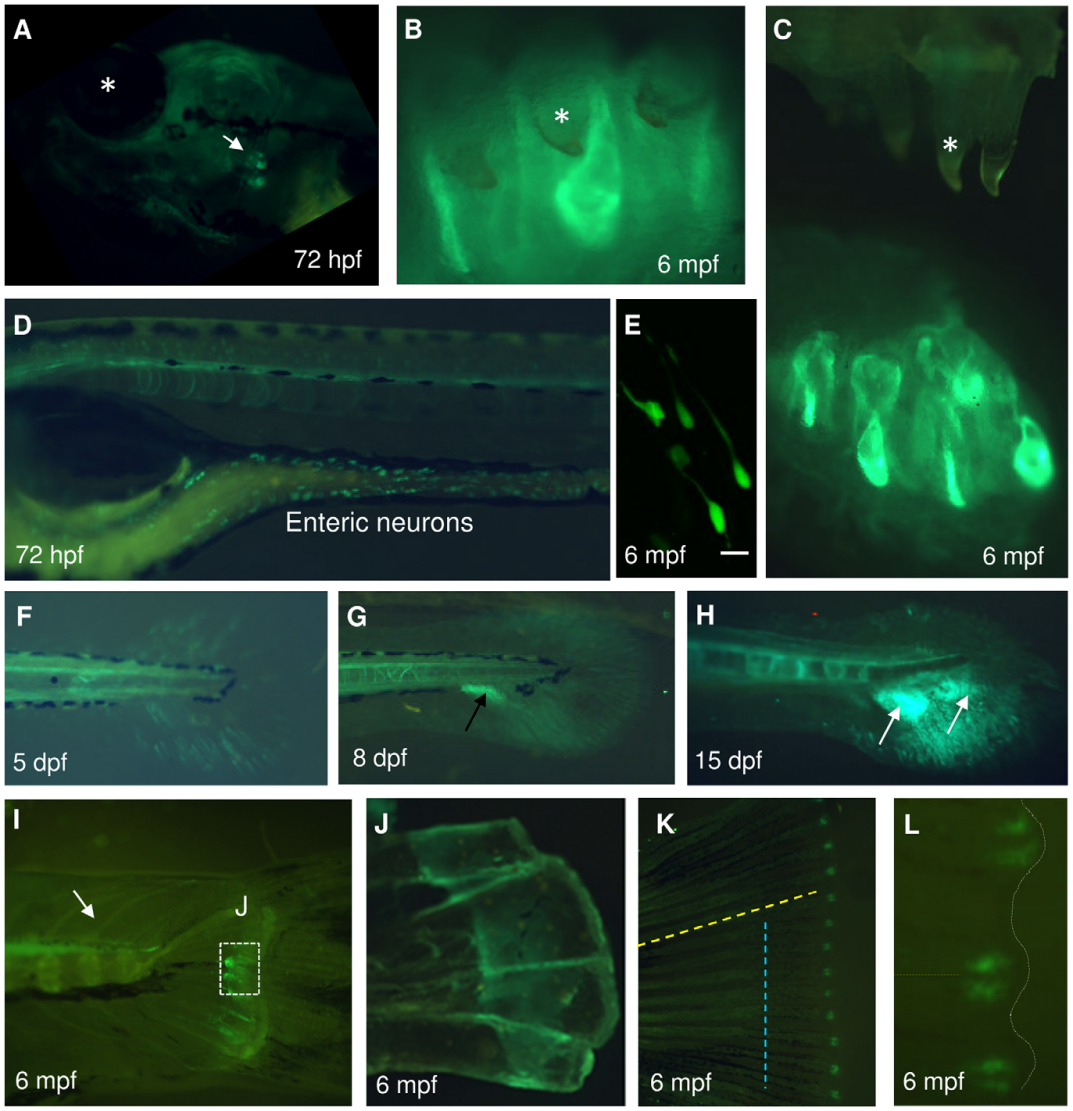
*Spon1b* expression in peripheral tissues of *Tg(spon1b:GFP).* **A.** *Spon1b* expression in pharyngeal teeth of zebrafish larvae at 3dpf (arrow). Asterisk: eye. **B-C**. Pharyngeal teeth in adult zebrafish with *spon1b:GFP* expressed in newly developing teeth. Asterisk: old mineralized teeth. Green signal: s*pon1b*-positive soft tissue. In C, soft tissue is moved off the hard tissue. **D**. *Spon1b:GFP* positive enteric neurons in intestine of developing larva, 72hpf (sagittal view of live specimen). **E.** *Spon1b:GFP* positive enteric neurons in adult zebrafish (confocal z-stack). **F-H.** Progression of *spon1b* expression in larval tail. Expression in larval fin rays at 5 dpf (F). Development of the adult tail primordium (arrow) by 8 dpf (G). Subsequent splitting into 2 domains (arrows in H, 15 dpf) that will give rise to the two adult caudal fin lobes. **I**. *Spon1b* expression in the endoskeleton of the adult zebrafish caudal fin. Note *spon1b*-positive axons of the motor neurons traversing the trunk and musculature (arrow). **J**. Dissected endoskeleton in (I), showing a thin sheet of *spon1b-*positive tissue covering the bone. **K.** *Spon1b* signal at the distal end of the growing adult tail 4 days post caudal fin amputation. Blue line shows the level of amputation and yellow line marks a growing lepidotrichium. **L.** High magnification image of region in (K) showing the two *spon1b*-positive domains of blastema, patterning each growing lepidotrichium (yellow line). White line: distal edge of regenerating caudal fin. Scale bars: 20 µm.

The zebrafish have only pharyngeal teeth, located posteriorly to the fifth pharyngeal arch, and these showed strong *spon1b:GFP* expression during development (Fig. 11A). The teeth, known to regenerate throughout the zebrafish lifespan [45], remained strongly labeled with the transgene in adult fish (Fig. 11B-C), and this was consistent with high *spon1b* mRNA levels determined by qPCR (data not shown). *Spon1b* expression was restricted to the soft tissue surrounding the mature teeth (Fig. 11C), which corresponds to the tooth-associated lamina and the replacement tooth developing from it [45].

The expression of *spon1b:GFP* in zebrafish enteric neurons was evident during early development (Fig. 11D). These cells, regulating gut motility, are located between the circular and longitudinal smooth muscle layers [46] and retain robust F-spondin expression throughout zebrafish life. An especially high density of *spon1b:GFP* positive neurons around the anal pore allowed us to easily observe them in live adult fish (data not shown). The distinct *spon1b:GFP* positive neurite extensions could be documented in adult gut using confocal microscopy (Fig. 11E).

*Spon1b* was also present in developing zebrafish fins. Early massive *spon1b* expression in the tail bud and caudal fin rays (Fig. 1C-E) was followed by a strong *spon1b:GFP* signal in the pectoral and abdominal fin buds in larval fish. The larval caudal fin in zebrafish is replaced by the adult fin through a well-characterized process [47]. Consistent with this, around 8 dpf *spon1b:GFP* expression was detected in the adult caudal fin primordium (Fig. 11F-H). Later, the primordium gives rise to the endoskeleton of the adult tail [47] and, in adult zebrafish, we documented a thin layer of cells covering the endoskeleton that continued expressing *spon1b* (Fig. 11I-J). Otherwise, in adult zebrafish, the caudal fin rays were devoid of *spon1b:GFP* expression.

Zebrafish are known to regenerate their caudal fin following partial amputation. This complex process involves reprogramming and cell migration, with extracellular matrix remodeling being essential for this process [48]. To determine whether F-spondin might be involved in fin regeneration, we conducted partial fin amputation and documented *spon1b:GFP* expression over a 2-week period of regeneration. There was no upregulation in *spon1b* expression during the first day post-trauma, which corresponds to the wound healing and epithelialization period. However, by the end of the second day of regeneration, qPCR measurements in fin tissue indicated initiation of *spon1b* mRNA production (data not shown). This coincided with the appearance of *spon1b:GFP* fluorescence at the distal ends of the fin rays (lepidotrichia) (Fig. 11K-L), in an area called the blastema, a zone of actively proliferating mesenchymal cells responsible for fin patterning and re-growth [49]. The fluorescence signal was typically restricted to paired domains at the tip of the growing rays of each lepidotrichium (Fig. 11L). *Spon1b* expression was observed in these areas throughout the fin regeneration process and disappeared after its completion.

## Discussion

The goal of the present study was to investigate F-spondin expression patterns in developing and adult zebrafish, in order to compare them to the regions of F-spondin expression reported in mammals, and to relate these patterns to the functional role of F-spondin suggested by *in vitro* studies. Unlike mammals, which have one F-spondin gene, zebrafish have two F-spondin homologs, *spon1a* and *spon1b*. Our systematic qPCR-based quantification of relative differences between these two homologs documented that *spon1b* expression is initiated much earlier, within hours post fertilization, and that mRNA abundance for *spon1b* in embryonic, larval or adult zebrafish tissues is consistently and substantially higher than that for *spon1a*. An earlier study also reported lower *spon1a* expression in zebrafish embryos, as determined by *in situ* hybridization [12]. We thus focused on characterizing the patterns of expression for *spon1b* and, for the first time, established a transgenic vertebrate model with stable *spon1b:GFP expression*. The localization of cells expressing the *spon1b:GFP* transgene in developing and adult zebrafish proved to be consistent with the areas highlighted by the complementary *spon1b in situ* hybridization method, with minor exceptions (see Results). The major advantage of the transgenic animal was that it permitted visualization of both the cell bodies and the projections originating from them.

Here we demonstrate that, following the onset of *spon1b* expression in structures involved in early patterning and polarization of zebrafish CNS, F-spondin-positive neurons are present in specific regions of the telencephalon, diencephalon, mesencephalon, hindbrain, and spinal cord. The projections of these F-spondin positive neurons can be local but, more often, extend between distant CNS regions, forming long neuronal tracts. The significance of F-spondin as a secretory ECM protein is highlighted by its expression in CSF-contacting cells with projections directed toward the ventricular walls. Importantly, the distribution of *spon1b* in adult zebrafish brain closely coincides with the proliferative zones, suggesting that F-spondin might be essential for adult neurogenesis. Use of this new transgenic model organism also allowed for the documentation of F-spondin expression patterns in the peripheral CNS, notably in enteric neurons, and in peripheral tissues involved in active patterning or proliferation in adults. These included the endoskeleton of zebrafish fins, as well as their continuously regenerating pharyngeal teeth. The latter result is notable given that F-spondin was recently found to be expressed in dental follicle cells of the developing tooth germ in mice [8], and in human periodontal cementoblasts, the activity of which is essential for mature tooth stability [7]. The role of F-spondin in actively proliferating tissues is further stressed by our observation of its *de novo* expression in the blastema, a critical area of cell proliferation and patterning formed during zebrafish fin regeneration. Together, these findings suggest multiple roles for F-spondin in the CNS and periphery of the developing and adult vertebrate and call for further investigations into the mechanisms of its actions.

### F-spondin Expression during the Segmentation Period

An important role of F-spondin during the segmentation period [22] is suggested by massive *spon1b:GFP* transgene expression in the fin buds, developing somites, optic primordia, floor plate and notochord around 10 hpf. This expression pattern is, generally, in agreement with previous *in situ* hybridization-based studies conducted in embryonic zebrafish, frog, chick, mouse and rat [4], [10]–[12]. The expression of *spon1b:GFP* in the developing forebrain and spinal cord neurons also suggests that F-spondin is involved in early patterning and polarization of the zebrafish CNS. This is consistent with *in vitro* studies showing that F-spondin can guide and promote the extension of neurites and axons of dorsal spinal [4], commissural [11] and hippocampal [13] cells in mammals, while inhibiting motor neuron outgrowth [50] and neural crest cell migration [51]. Overall, the visualization of *spon1b:GFP* accumulation in specific areas of the developing zebrafish embryo allows for earlier detection of *spon1b*-producing cells. This might explain prior ISH-based reports of more restricted *spon1b* expression during early zebrafish development [12].

### F-spondin-expressing Neurons form Long Projections

In zebrafish CNS, F-spondin expression appears to be limited to neurons, indicated by consistent co-localization with Hu C/D. Many of these neurons extend long projections, including RGC axons to the TeO, medial and lateral olfactory tracts (MOT and VOT), and sensory and motor neurons of the midbrain, hindbrain and spinal cord. Moreover, F-spondin expression highlights all the components of the long dorsal conduction pathway (DCP) [31], with an especially strong *spon1b:GFP* signal in the FR, extending from the Hb in the dorsal diencephalon to the NIn and SR of the ventral tegmentum. Together, this expression in adult brain implies that F-spondin function is not limited to the initial patterning and direction of long axons, but that it may serve as a short-range cue that remains associated with the cell producing it, promoting maintenance of its long axon and synaptic connections. Similar roles have been previously suggested for other ECM proteins, including Reelin [52], [53].

### F-spondin Expression in Circumventricular Regions and CSF-contacting Neurons

F-spondin is reported to be a diffusible signaling molecule [5]. Consistent with this, we observed its expression in several regions that contain CSF-contacting neurons. These cells have two principal functions. Through their dendrites, they can receive information about chemical content, pressure or flow of the CSF, then convey it to various brain regions via their axons that terminate not only in the periventricular areas but as far away as the telencephalon or the spinal cord [54]. Alternatively, the axons of the CSF-contacting neurons can produce and release neurotransmitters, peptides and other biologically active molecules into the CSF, providing chemical signals to other brain areas lining the ventricular spaces. Accordingly, we find that F-spondin positive neurons located along the mediobasal part of the diencephalic ventricle (Hv) form distinct tracts directed away from the ventricle, thus potentially serving as chemosensory neurons. This is in contrast to other CSF-contacting cells that extend their F-spondin positive axons into the ventricular lumen and are likely to contribute soluble F-spondin protein to the CSF. Especially rich in such cells are the nuclei positioned along the wall of the diencephalic ventricle and its recesses, including the preoptic nuclei, the paraventricular organ, and the nucleus of the posterior recess (NPR) of the caudal hypothalamus (Fig. 7). The two latter nuclei are known to be strongly labeled for several catecholaminergic markers in zebrafish [55], indicative of serotonergic [56] and dopaminergic [57] activity. Other magnocellular neurosecretory neurons in this nucleus in lower vertebrates, including fish, show immunoreactivity to neurophysin, vasotocin, isotocin/mesotocin, somatostatin or enkephalin antibodies [54]. Together, this raises an important question as to which neurochemical systems may regulate F-spondin release into the CSF, and which ones might be affected by the presence of F-spondin.

The medullo-spinal component of the CSF contacting system, which extends throughout the rhomboencephalic ventricle and central canal [54], also expresses F-spondin mRNA. The neurons of this area extend stereocilia into the CSF, which act as mechanoreceptors when they contact Reissner’s fiber [54]. This enigmatic thread-like glycoprotein fiber, spanning the entire length of the central canal in the majority of vertebrates, is produced largely by the subcommissural organ (SCO), with some contribution from other circumventricular areas [21]. An earlier study in embryonic zebrafish reported the expression of F-spondin mRNA in the SCO (*in situ* hybridization) and the presence of *spon1b* protein in Reissner’s fiber (immunohistochemistry) [12]. In contrast, we could not detect *spon1b* in the SCO in developing or adult animals with the methods used here. Nevertheless, the *spon1b:GFP* transgene highlighted the flexural organ, which is also a source of Reissner’s fiber-forming proteins [21] and the habenula, located immediately dorsal to the posterior commissure and SCO.

Indeed, among the circumventricular organs (CVO) identified in zebrafish [58], the Hb nuclei attracted our special attention due to the early onset and exceptionally robust nature of *spon1b* expression in this important structure. Although the CSF-contacting properties of Hb neurons have not yet been characterized in zebrafish, evidence collected in other species suggests that Hb nuclei may contain actively secreting mast cells (e.g., in doves) [59] and may receive afferent projections from the CSF-contacting PVO via the FR (e.g., in lungfish) [54]. Using *in situ* hybridization, the expression of F-spondin was detected in the dorsal Hb nuclei of developing zebrafish in some [29] though not other [12] studies. We have also documented broad F-spondin expression in Hb of adult zebrafish using an *in situ* hybridization approach and the same probe used in an earlier study that demonstrated the presence of F-spondin in Hb [60]. Although the *spon1b:GFP* transgene showed prominent expression in Hb starting early in development, its localization was more restricted than that suggested by *in situ* hybridization, highlighting only laterally-positioned Hb nuclei in larval fish. Thereafter, the transgene allowed for detailed *in vivo* tracing of the migration of the two lateral Hb nuclei toward the midline during maturation, until they finally acquired their rostro-ventral location, corresponding to the ventral Hb nucleus (vHb) in adult zebrafish. This is consistent with a recent report on the complex migratory path of the lateral Hb nuclei during zebrafish ontogenesis and the identification of the lateral Hb in mammals as being homologous to the lateral Hb in larval zebrafish but the ventral Hb in adult zebrafish [28].

In addition to the distinct ventral and dorsal nuclei of the Hb, the dorsal Hb in zebrafish is further divided into the dorso-lateral (dlHb) and dorso-medial (dmHb) sub-divisions, indicated by distinct gene expression patterns and projections to the dorsal and ventral NIn, respectively [29], [30], [61]. In other species, it has been suggested that the Hb has even more complex morphological and functional structure [62], [63]. Our data, as well as others’, suggest that the Hb in zebrafish is also likely to have more than three subdivisions [29], [30], since the *spon1b:GFP* transgene, in addition to vHb, highlighted a previously undescribed subnucleus. We named it the inferior subnucleus of the dorso-medial Hb (dmHbi) based on its localization, cell morphology and projections to ventral NIn, consistent with dmHb. However, the dlHb and dmHb are known to keep a midline position starting with early development [60], [64], while we do not detect a *spon1b:GFP* signal in the midline region until maturation occurs. It is thus possible that dmHbi starts expressing the transgene only later in life, in contrast to the early onset of *spon1b* production in the lateral, later turned ventral, Hb. Overall, in view of the major role that the Hb plays in emotional and cognitive functions in diverse species, including humans [65], specific mapping of *spon1b:GFP* transgene expression here could shed light on the role of the lateral Hb in CNS development and function throughout vertebrate life.

### F-spondin Expression in Neurogenic Zones

During embryonic stages in vertebrates, ECM proteins are abundant in the developing brain. Later, in adults, they become more restricted to brain regions with germinal capacities [66]. For example, the rostral migratory stream (RMS) and granular zone of adult rodents, regions abundant in neural stem cells, are also characterized by high expression of ECM proteins [67]. In particular, F-spondin is present in the RMS of mice and was suggested to be part of the signaling cascade for chain formation, migration, and detachment of neuroblasts from the stream via its interaction with the ApoER2 receptor and intracellular adapter Dab1 [18]. Here, we report that *spon1b* in zebrafish is abundant not only in the areas that resemble mammalian neurogenic niches (i.e., Vv as RMS [68]), but also in all other proliferative zones along the rostro-caudal axis of the zebrafish brain. This suggests that the role of F-spondin in neurogenesis might be broad and not specific to the telencephalic niches. *In vitro* studies in mammalian hippocampal and cortical progenitor cell lines have identified F-spondin as a secreted protein that regulates migration and differentiation of neurons [5]. A potential role for F-spondin in these processes is also supported by our data showing that the habenular and cerebellar niches, which contain the largest migratory stem cells populations in zebrafish [41], are also the regions that contain the highest density of F-spondin-expressing cells (in Hb) or fibers (in CCe).

Double staining for *spon1b:GFP* and the proliferative marker BrdU revealed that, in all of the stem cell niches examined, the newly divided cells do not express *spon1b*. Even a month later, the BrdU-positive cells that migrate to the *spon1b*-rich areas, e.g., vHb, remain *spon1b* negative. Nevertheless, we could observe a few cells positive for both BrdU and *spon1b:GFP* that migrated to the tegmentum within a month after cell division, and were in the process of extending long projections. Still to be elucidated is the extent to which F-spondin is involved in adult neurogenesis, and the mechanism through which this ECM protein could contribute to the microenvironment of the neurogenic niches, the migration of new cells, and their ability to form long projections.

### The Role of *F-spondin* in Peripheral Tissues

In addition to the proliferative niches in the brain, the peripheral tissues involved in patterning of newly forming or regenerating structures express *spon1b* throughout the life of the zebrafish. These include the endoskeleton of the zebrafish fins, involved in patterning, and the unique blastema region of fin rays undergoing regeneration following partial amputation. Moreover, quite remarkable is the massive and constant expression of *spon1b* in the soft lamina surrounding the pharyngeal teeth, which continuously renew throughout zebrafish life. In fact, recently F-spondin was identified as being important for mammalian tooth development and maintenance, and its specific expression in the dental follicle cells of the tooth germ in mice [8], and in human periodontal cementoblasts, essential for the stabilization of the periodontal ligament and the tooth itself [7]. Similarly, the presence of F-spondin in embryonic chick and rat cartilage and its ability to stimulate chondrocyte activity and collagen degradation in cultured cartilage explants, [6] suggest additional F-spondin functions.

The peripheral nervous system arises from neural crest cells that migrate to different areas of the organism. F-spondin has been found to be involved in patterning of the sympathetic nervous system by acting as a signaling molecule that prevents neural crest cells from entering the caudal end of each somite [50]. Our finding that F-spondin is expressed in enteric neurons of zebrafish, raises the interesting question of whether it might also play a role in enteric neuron differentiation, migration, or maintenance, in other vertebrates, including humans, and calls for further investigation of this issue.

Together, our findings imply multiple roles for F-spondin in the CNS and periphery of the developing and adult vertebrates, making the zebrafish an attractive model to investigate the role of this ECM protein in normal and pathological conditions.

## Methods

### Animal Care and Maintenance

Adult male and female zebrafish (*Danio rerio*, AB wild type strain, 5 fish/3-L tank) were housed in a 14 h light/10 h dark cycle, in a temperature (26.5°C) and pH (7.0–7.4) controlled multi-tank re-circulating water system (Aquaneering, San Diego, CA, USA). Animals were fed three times a day with live brine shrimp (Brine Shrimp Direct, Ogden, Utah. USA), enriched with fish pellets (Lansy NRD, Salt Lake City, UT. USA). Embryos were raised at 28.5°C in a 12 h light/12 h dark cycle before being transferred to system tanks at 21 dpf.

All animal procedures were performed in accordance with the protocol approved by the Institutional Animal Care and Use Committee (IACUC) at Boston University School of Medicine.

### Generation of Transgenic Zebrafish

A plasmid with the enhanced green ffiuorescent protein (EGFP) under the control of the zebrafish *spon1b* promoter (Accession# NM_131517) was constructed. The recombinant *spon1b:GFP* construct was obtained by a combination of assembly PCR and traditional cloning. By PCR, using the appropriate primers, a 10.3-kb DNA sequence from the 5′ promoter region of the *spon1b* gene, obtained from the genomic CH211-20O6 clone (BACPAC Resources Center, Children’s Hospital Oakland Research Institute, Oakland, CA), and a 0.86-kb fragment containing the 3′ flanking region of *spon1b*, were placed upstream and downstream, respectively, of the EGFP sequence. The resulting 12.2-kb construct was transferred into a modified pNEB193 plasmid containing two meganuclease I-SceI sites. The one-cell stage zebrafish embryos were co-injected with the plasmid DNA containing the construct and the meganuclease I-SceI, as recommended [69]. Injected embryos were screened for the presence of GFP fluorescence between 48 and 72 hpf. Positive embryos were raised to sexual maturity and out-crossed with wild type fish to identify transgenic carriers. The presence of green fluorescence in the F1 offspring identified transgenic founder zebrafish. Out of 69 adult GFP-positive fish, two had germ-line transmission of *spon1b:GFP*, with similar expression patterns in transgenic F1 progeny. The line that had the stronger fluorescence signal was used for the studies described here.

### Real-time Quantitative Reverse-Transcriptase PCR (qPCR)

Total RNA was extracted and purified from whole embryos at desired stages and adult brains using RNAeasy Qiagen (Qiagen, Chatsworth, CA, USA), according to the manufacturer's protocol. RNA extraction from brain tissue was conducted using QIAzol Lysis Reagent (Qiagen, Chatsworth, CA, USA) before RNA isolation using RNAeasy Qiagen kit. The quantity and quality of RNA was determined spectrophotometrically, at 260 nm and 260/280 nm. The same amount of RNA from each sample was converted into cDNA using the High-Capacity cDNA Archive Kit (Applied Biosystems, Foster City, CA, USA), according to the manufacturer's instruction. qPCR was performed using a TaqMan® Universal PCR Master Mix and ABI Prism 7300 Real Time PCR System (ABI, Foster City, CA, USA). The TaqMan® primers and probes {5′ FAM, 3′ TAMRA} for spon1a and spon1b were designed based on previously reported sequences of zebrafish genes and obtained from ABI: spon1a Forward, 5′-GTGACCAAGAGGAGGATTATGCT-3′, Reverse, 5′-TGTCATGAATTGCGTGTCTTCCT-3′; {ATCGATTATCTGGAAATTT}, spon1b Forward, 5′-GAAGGAGAGCCAGAAACTTACCAA -3′, Reverse, 5′-CAATGAGGGTAAAACCACGAAAGT-3′; {CACCTACAGAGTGAGTTTG}, and β-actin Forward, 5′-GCTGTTTTCCCCTCCATTGTTG -3′, Reverse, 5′-TTTCTGTCCCATGCCAACCA-3′; {CCCAGACATCAGGGAGTG}. Gene expression was normalized using β-actin expression level for each individual fish sample. A second housekeeping gene, EGF-1, was also used in some of the assays to control for lack of non-specific changes in mRNA abundance Forward, 5′-TCCTTGCGCTCAATCTTCCAT -3′, Reverse, 5′-GCACGGTGACAACATGCT-3′; {ACCAGCCCATGTTTGAG}. Relative mRNA expression level was calculated using the standard comparative delta-Ct method. Within each experiment, embryonic or adult brain tissue samples were collected and processed in parallel, and the expression was measured within the same microplate, each sample in triplicate.

### Tissue Preparation for RNA and Protein Staining

To obtain tissue samples, adult zebrafish were anesthetized using tricaine (MS-222, Sigma). When they stopped breathing, they were decapitated and the heads fixed in 4% paraformaldehyde in 0.1 M phosphate buffer (PFA) for at least 2 hours. Then, the brains were dissected out and re-fixed in 4% PFA overnight. The following day, the brains were cryopreserved in a solution of 30% sucrose until they sank. Then, they were fast frozen in isopentane, embedded in OCT (Tissue-Tek, Torrance, CA, USA), and serially cryosectioned in the coronal plane every 20 µm. Sections were stored at −80°C until processed for *in situ* hybridization or immunohistochemistry. Embryos were collected without removing the chorion, at different developmental stages, and fast frozen in liquid nitrogen for qPCR analysis.

### *In situ* Hybridization

The plasmid containing the sequence for *spon1b* was kindly provided by Drs. Halpern and Gamse. After digestion with EcoRI, *in vitro* transcription was conducted using T7 RNA polymerase and digoxigenin labeling mix to generate antisense RNA probes. For *in situ* hybridization, the sections were brought to room temperate (RT) for 45 minutes. Then they were rehydrated in PBS 2 x 10 minutes. After triethanolamine/acetic anhydride treatment for 10 minutes, the sections were permeabilized in 1% Triton X-100/PBS for 30 minutes and washed with PBS. Then, the slides were pre-hybridized for 2 h at RT in a solution containing 10% of 50%-Dextran sulfate (American Bioanalytical), 20% 20X SSC buffer (Ambion), 50% formamide (American Bioanalytical), 2% 50X Denhardt’s (Sigma), 5% fish sperm DNA (Invitrogen) and 15% DEPC water, followed by overnight hybridization with DIG-labeled RNA probes (0.2 ng/ul) at 65°C. The next day, the sections were incubated in 0.2X SSC at 65°C for 2 hours, followed by 2 more washes of 0.2XSSC at RT for 10 min each. Sections were blocked in 2% normal sheep serum in TBST for 1 hour at RT, and then incubated with anti-DIG alkaline phosphatase conjugate (1∶7000, Roche) overnight at 4°C. The third day, the sections were washed with TBST 3×10 minutes, and incubated in color reaction buffer (0.1 M Tris, 0.1 M NaCl, 0.05 MgCl2, 0.05% Tween-20) for 10 minutes. Then, the signal was visualized with BM Purple substrate solution (Roche) according to the manufacturer’s protocol, and the reaction stopped with PBS.

Whole-mount *in situ* hybridization was performed essentially as previously described (Musson et al., 2009). Briefly, specimens were rehydrated, digested with Proteinase K, pre-hybridized for 2 hours at 70°C in solution containing 50% formamide, 5× SSC buffer, 1% sodium dodecyl sulfate, 50 mg/ml yeast RNA and heparin, followed by overnight hybridization with DIG-labeled RNA probes (0.2 ng/µl). Samples were then incubated with anti-DIG alkaline phosphatase conjugate overnight at 4°C. Signal was visualized with centrifuged BM Purple substrate solution (Roche Diagnostics), according to the manufacturer’s protocols.

### Immunohistochemistry

Immunohistochemical procedures were performed following standard protocols [70]. In brief, sections were brought to RT and rehydrated in 0.1M KPBS. Cryosections were blocked in 5% Normal Donkey Serum in KPBS for 60 min (only for double fluorescent immunostaining), and incubated in primary antibodies diluted in 0.4% Triton-X100/KPBS for 48 h at 4°C. Then, sections were washed and incubated in secondary antibodies diluted in 0.4% Triton-X100/KPBS for 1 hour at RT. For double immunostaining experiments, sections were washed before repeating the same procedure with the following antibody. Slides were coverslipped using Immuno-mount (Vector). Modifications to the protocol: For HuC/D, sections were boiled for 30 min in 1 M TBST after rehydration. For BrdU, slides were pretreated with 50% formamide/50% 2X SSC at 65°C for 2h, followed by 2 M HCl at 37°C for 45 minutes and washed with boric buffer pH 8.5 and KPBS for 10 min, according to Zupanc *et al.* [42]. The following primary antibodies were used: goat anti-GFP (1∶500000 for chromogenic staining and 1∶5000 for fluorescent staining, Abcam); mouse anti-Hu C/D (1∶100, Invitrogen), rat anti-BrdU (1∶1000, Abcam), rabbit anti-GFAP (Abcam). The following secondary antibodies were used: donkey anti-Goat Alexa 568 (1∶1000, Invitrogen), donkey Anti-Rat Alexa 488 (1∶1000 Invitrogen), donkey anti-Rabbit (1∶2000 Invitrogen), rabbit anti-goat biotin (1∶1000, Vector), and rabbit anti-mouse Biotin (1∶1000, Vector). Chromogenic visualization was done with the Standard ABC Elite Kit (Vector) with NiDAB as a substrate. Fluorescent visualization was done with appropriate secondary antibodies labeled with Alexa 568 or, in the case of Hu C/D, with amplification by streptavidin Alexa 488 (Invitrogen).

### Imaging

Screening of live animals and freshly cut brain tissue was carried out using a Leica dissecting fluorescence microscope. For detailed *in vivo* analysis of *spon1b*:GFP expression patterns at different stages of zebrafish development, embryos or larvae were embedded in 1% low-melting point agarose (Shelton Scientific) in the desired orientation, and covered by egg-water after solidification. Fluorescent images were taken in an inverted Zeiss Axiovert 200M LSM 510 confocal laser-scanning system (Zeiss, Thornwood, NY) through the dry 20X and the water-immersion 40X objective lenses. The Z-sections were taken at an optical slice of 1–2 µm. Confocal settings were optimized to control for signal crossover. Detector gain and amplitude offset were set to maximize the linear range without saturation and were kept consistent throughout experiments. Images were stacked, composites were generated, and co-localization was determined by means of orthogonal slice analysis of each section using the LSM 510 software.

### Nomenclature

Sections were analyzed in comparison to the Atlas of the Neuroanatomy of the adult zebrafish brain [71]. Neuroanatomical designations follow mostly those of Wullimann and colleagues [71] complemented by additional studies on BNSM [27], NPR [32], TeO layers [34], and Hb [28].

## Abbreviations

A: Anterior hypothalamic nucleus
APP: Amyloid precursor protein
BNSM: Bed nucleus of stria medullaris
BrdU: 5-Bromo-2-deoxyuridine
DAB-1: Disabled-1
Cans: Ansulate commissure
Cant: Anterior commissure
CC: Cerebellar crest
CCe: Cerebellar corpus
Cgus: Gustatory commissure
cHb: Caudal habenula
Chor: Horizontal commissure
CM: Mammillary corpus
Cp: Central posterior thalamic nucleus
Cpost: Posterior commissure
Ctec: Tectal commissure
D: Dorsal pallium
Dd: Dorsal nucleus of dorsal telencephalon
dHb: Dorsal habenula
Dl: Lateral nucleus of dorsal telencephalon
dlHb: Dorsolateral habenula
Dm: Medial nucleus of dorsal telencephalon
dmHb: Dorsomedial habenula
dmHbi: Inferior nucleus of dorsomedial habenula
DOT: Dorsal optic tract
DON: Descending octaval nucleus
Dp: Posterior zone of dorsal telencephalic area
ECM: Extracellular matrix
EG: Granular eminence
fp: Floor plate
FR: Fasciculus retroflexus
Fv: Ventral funiculus
GL: Glomerular layer of olfactory bulbs
H: Hypothalamus
Hb: Habenula
Hc: Caudal hypothalamus
Hd: Dorsal hypothalamus
Hv: Ventral zone of periventricular hypothalamus
IAF: Internal arcuate fibers
ICL: Inner cell layer of olfactory bulbs
LLF: Lateral longitudinal fasciculus
LOT: Lateral olfactory tract
LX: Vagal lobe
Me: Medula oblongata
MLF: Medial longitudinal fasciculus
mn: Motor neuron
MOT: Medial olfactory tract
NI: Nucleus isthmi
Nin: Interpeduncular nucleus
nMLF: Nucleus of medial longitudinal fasciculus
no: Notochord
NPR: Nucleus of the posterior recess
NXm: Motor nucleus of vagus
OB: Olfactory bulb
on: Optic nerve
PGm: Medial preglomerular nucleus
PGl: Lateral preglomerular nucleus
PGZ: Periventricular grey zone
PNT: Posterior tuberal nucleus
Ppa: Parvocellular preoptic area, anterior
Ppp: Posterior preoptic area
PVO: Paraventricular organ
PM: Magnocellular preoptic nucleus
PTN: Posterior tuberal nucleus
PPd: Periventricular pretectal nucleus, dorsal part
PPv: Periventricular pretectal nucleus, ventral part
Pur: Purkinje cell layer
PVO: Paraventricular nucleus
SAC: Stratum album centrale
SCO: Subcommissural organ
SFGS: Stratum fiibrosum et griseum superficiale
SGC: Stratum griseum centrale
SGN: Secondary gustatory nucleus
sm Stria medullaris
SM: Stratum marginale
SO: Stratum opticum
SPV: Stratum periventriculare
SR: Superior raphe
RGC: Retinal ganglion cells
RF: Reticular formation
RV: Rhomboencephalic ventricle
Tel: Telencephalon
TeO: Optic tectum
TPp: Posterior tuberculum
TL: Torus longitudinalis
TTB: Tectobulbar tract
URL: Upper rhombic lip
Val: Valvula cerebellis
Vl: Ventrolateral nucleus of the thalamus
VOT: Ventrolateral optic tract
Vv: Ventral nucleus of ventral telencephalic area
vHb: Ventral nucleus of habenula
vNIn: Ventral interpeduncular nucleus
Te: Telencephalon
